# Short Message Service Reminder Nudge for Parents and Influenza Vaccination Uptake in Children and Adolescents With Special Risk Medical Conditions: The Flutext-4U Randomized Clinical Trial

**DOI:** 10.1001/jamapediatrics.2022.6145

**Published:** 2023-02-20

**Authors:** Jane Tuckerman, Kelly Harper, Thomas R. Sullivan, Alana R. Cuthbert, Jennifer Fereday, Jennifer Couper, Nicholas Smith, Andrew Tai, Andrew Kelly, Richard Couper, Mark Friswell, Louise Flood, Christopher C. Blyth, Margie Danchin, Helen S. Marshall

**Affiliations:** 1 Adelaide Medical School, The University of Adelaide, Adelaide, South Australia, Australia; 2 Vaccine Uptake Group, Murdoch Children’s Research Institute, Royal Children’s Hospital, Melbourne, Victoria, Australia; 3 Faculty of Medicine, Dentistry and Health Sciences, University of Melbourne, Melbourne, Victoria, Australia; 4 Women's and Children’s Health Network, North Adelaide, South Australia, Australia; 5 Robinson Research Institute, The University of Adelaide, Adelaide, South Australia, Australia; 6 SAHMRI Women and Kids, South Australian Health and Medical Research Institute, Adelaide, South Australia, Australia; 7 School of Public Health, The University of Adelaide, Adelaide, South Australia, Australia; 8 Communicable Disease Control Branch, South Australian Department of Health and Wellbeing, Adelaide, South Australia, Australia; 9 Wesfarmers Centre of Vaccines and Infectious Diseases, Telethon Kids Institute, University Western Australia, Perth, Western Australia, Australia; 10 Department of Infectious Diseases, Perth Children's Hospital, Perth, Western Australia, Australia; 11 School of Medicine, University of Western Australia, Perth, Western Australia, Australia; 12 Department of Microbiology, PathWest Laboratory Medicine WA, Perth, Western Australia, Australia; 13 Department of General Medicine, The Royal Children’s Hospital, Melbourne, Victoria, Australia

## Abstract

**Importance:**

Children with chronic medical conditions are at increased risk of severe influenza. Uptake of influenza vaccination in children and adolescents with these identified special risk medical conditions (SRMCs) is suboptimal.

**Objective:**

To assess the effectiveness of Flutext-4U, a parent short message service (SMS) reminder nudge intervention, in increasing influenza immunization in children and adolescents with SRMCs.

**Design, Setting, and Participants:**

This randomized clinical trial was conducted at a tertiary pediatric hospital in Adelaide, South Australia, from April 15 to September 30, 2021. Children and adolescents aged 6 months to younger than 18 years with SRMCs and a subspecialist outpatient appointment over a 5-month period during the Australian seasonal influenza vaccination season (April-August 2021) were eligible to participate. Follow-up was until September 30, 2021.

**Interventions:**

Participants were randomly assigned (1:1 ratio) to control: clinician nudges (hospital vaccine availability, ease of access, and recommendation from hospital subspecialists) or SMS intervention (control conditions plus an additional SMS reminder nudge to parents), with randomization stratified by age group (<5 years, 5-14 years, or >14 to <18 years).

**Main Outcomes and Measures:**

The primary outcome was influenza vaccination, as confirmed by the Australian Immunisation Register.

**Results:**

A total of 600 participants (intervention group: 298 [49.7%]; mean [SD] age, 11.5 [4.6] years; 162 female participants [54.4%]; control group: 302 [50.3%]; mean [SD] age, 11.4 [4.7] years; 155 female participants [51.3%]) were included. Influenza vaccination was 38.6% (113 of 293) in the SMS intervention group compared with 26.2% (79 of 302) in the control group (adjusted odds ratio [aOR], 1.79; 95% CI, 1.27-2.55; *P* = .001). Time to vaccine receipt was significantly lower among SMS participants (adjusted hazard ratio, 1.67; 95% CI, 1.25-2.22; *P* < .001). For participants randomly assigned by June 15, a significantly greater proportion receiving the SMS intervention were vaccinated during the optimal delivery period April to June 30 (SMS group: 40.0% [76 of 190] vs 25.4% [50 of 197]; aOR, 1.97; 95% CI, 1.28-3.06; *P* = .002).

**Conclusions and Relevance:**

Results of this randomized clinical trial suggest that an additional SMS reminder nudge for parents delivered in the tertiary care hospital setting to children and adolescents with SMRCs resulted in higher influenza vaccine uptake compared with clinician nudges alone.

**Trial Registration:**

ANZCTR Identifier: ACTRN12621000463875

## Key Points

**Question:**

What is the incremental effectiveness of providing a parent short message service (SMS) reminder nudge in addition to a clinician nudge alone (control) for children and adolescents with special risk medical conditions to improve influenza vaccine receipt?

**Findings:**

In this randomized clinical trial of 600 children and adolescents, a parent SMS reminder nudge increased vaccination uptake.

**Meaning:**

Results suggest that parent-clinician nudges are more effective for optimizing influenza vaccine receipt than clinician nudges alone.

## Teaser

This randomized clinical trial investigates the effectiveness of a parent short message service (SMS) reminder nudge to improve influenza vaccination uptake in children and adolescents with special risk medical conditions.

## Introduction

Children and adolescents with special risk medical conditions (SRMCs) are a priority group for influenza immunization because of their increased risk of influenza-associated hospitalization, intensive care admission, mechanical ventilation, and death.^1,2,3^ Similar to other countries internationally, approximately one-half of all children hospitalized with influenza in Australia have at least 1 SRMC.^2,4,5,6,7,8^ Influenza vaccination can reduce the risk of influenza-associated hospitalization in children by 65% to 70%, especially for children at increased risk.^9,10^ Despite available funding from the Australian National Immunisation Program for children with SRMCs to receive the vaccine annually,^8^ coverage is inadequate, with only 40% to 52% uptake across Australia.^11,12,13^

Many reasons for low influenza immunization rates in children with SRMCs are modifiable,^12,14,15,16,17,18,19,20,21^ and barriers include lack of access to immunization services, lack of health care professional (HCP) recommendation or negative recommendation, safety concerns, competing priorities, and lack of knowledge of the recommendation.^11,12,13,22^ Children are more likely to receive the influenza vaccine if their parents recall the recommendation from their child’s specialist.^11^ However, less than 58% of parents recall their child’s pediatrician making a recommendation for influenza immunization.^11,13^

Several barriers to influenza vaccination exist at the clinician level. Some HCPs may not have the knowledge and/or confidence to determine at-risk conditions. Specialists may not feel responsible for recommending primary care interventions, such as vaccination.^23^ Some hospitals have services providing immunization free of charge to children with SRMCs; however, many hospitals and HCPs recommend that children visit their family physician or general practitioner (GP) for immunization, adding to the burden of health care visits for these families.

One promising approach to promote desirable behavioral choices, including obtaining the influenza vaccine, is to make subtle changes to the environment in which individuals make decisions; this approach is termed *choice architecture*. When choice architecture is designed to predictably influence behavior without restricting choice, it is often called a *nudge*.^24^ Nudges are built on the principle that human decision-making is often based on automatic rather than deliberate thought processes alone and have been successfully used in other disciplines to shift behavior toward better health choices.^25,26^ A key concept of the nudge is to align the environment within which a choice is made to optimize positive behavior change.^27^ For vaccination, nudges are likely to act on a person’s motivation, and they serve as a cue to action, or they may influence clinician behavior, such as requiring a clinician to actively choose to accept or cancel an order for the influenza vaccine.^28^ In a systematic review, the level and direction of a nudge’s association with vaccination has been shown to vary context,^29^ with recent studies demonstrating that a nudge’s content as well as the timing of its delivery can influence impact.^30,31^

For children with SRMCs, interventions targeting practices and parents or children have been shown to increase influenza vaccine coverage.^32^ Text message or short message service (SMS) reminders sent by HCPs are a low-cost alternative and have been shown to increase vaccine uptake in some at-risk groups.^33,34,35,36^

We developed a nudge intervention, Flutext-4U, which included a parent-level nudge. An SMS reminder was sent to the child’s primary carer, which is hereafter referred to as parent (on behalf of the hospital). It is a practical and needs-based approach, providing information and reminders concurrently. Clinician-level nudges were incorporated into standard practice. Prompt/reminder stickers and medical record bookmarks were placed at the relevant clinical notes page for notes entry by the clinician, which were meant to assist hospital specialists and to facilitate vaccine recommendation. All elements were centrally coordinated. This article reported on the evaluation of the SMS (parent nudge) intervention.

## Methods

### Study Design

This was a parallel-group randomized clinical trial (RCT) conducted from April 15 to September 30, 2021, of the SMS intervention on receipt of influenza vaccine in children with SRMCs. The trial was approved by the Women’s and Children’s Health Network (WCHN) human research ethics committee, with a waiver of informed consent approved for parents to participate in this trial. A waiver of consent was used as knowledge of the SMS intervention would likely have influenced vaccine uptake in control group participants. Additional data on the acceptability of the SMS intervention were collected via parent survey, with consent implied by completion. No changes to study design or the protocol^37^ were made after study commencement. The trial protocol and statistical analysis plan are provided in Supplement 1 and Supplement 2, respectively. This study followed the Consolidated Standards of Reporting Trials (CONSORT) reporting guidelines.

### Study Location

The study was conducted at the Women’s and Children’s Hospital (WCH), 1 of 3 public pediatric and obstetric hospitals across metropolitan Adelaide, South Australia. The 295-bed hospital is the jurisdiction’s leading provider of specialist care for children; it includes care for all the pediatric specialties and has the largest obstetric service. There are more than 30 000 admissions, 250 000 outpatient appointments, and 5000 births annually.

### Participants

#### Inclusion Criteria

Children and adolescents (aged 6 months to <18 years) with SRMCs were identified from the WCH’s outpatient department appointment lists. Eligibility screening was completed by pediatric specialists using criteria set out in the National Immunisation Program^8^ for funded influenza vaccination. Exclusion criteria were^1^ previous receipt of the 2021 influenza vaccine documented on the Australian Immunisation Register (AIR),^2^ a younger sibling to an already enrolled participant (to ensure parents were not randomized twice),^3^ no listed mobile phone number for parent,^4^ and a diagnosis of cystic fibrosis, as these children receive additional influenza vaccine delivery support and messaging within the WCH environment.

### Randomization and Blinding

An independent statistician (not otherwise involved in the trial) prepared the randomization schedule. Parents were randomly assigned to study arm in a 1:1 ratio using ralloc.ado, version 3.7.6 in Stata, version 16 (StataCorp). Allocations were performed using randomly permuted blocks of size 4, stratified by child age group (<5 years, 5-14 years, >14 to <18 years). The schedule was provided electronically to the support staff of WCHN’s information communication technology applications system. The support staff then allocated participants according to the schedule. The trial statistician (A.R.C.) remained blinded until the database was locked for analysis. Given the nature of the intervention, the study coordinator (K.H.) was not blinded to allocation. Intervention components were provided, as per study trial arm.

### Control

In the control arm, influenza vaccine signage was placed around the hospital, and vaccine availability and ease of access were ensured. Clinician-level nudges comprised influenza vaccine reminder stickers on the front of hard-copy case notes and bookmarks at the relevant clinical notes page. The purpose of these nudges was to assist hospital specialists to make a vaccine recommendation during the appointment to the parent(s) of all children in the trial.

### Intervention

The intervention comprised a parent-level nudge, in addition to clinician nudges. In the parent nudge, SMS reminders were sent to the child’s parent (using MessageMedia software) in a nondirective educational approach advising them that their child/adolescent was eligible for a funded influenza vaccine and that they could receive it at the WCH immunization clinic or by their GP. The SMS read as follows:

“Influenza may be serious for children with specific medical conditions. The Women’s and Children’s Hospital recommends the influenza vaccine for all medically at-risk children. *NAME* is recommended to receive the influenza vaccine. The vaccine is free and available at GP clinics or at the WCH. Ask at your upcoming WCH appt. If your child has already received the 2021 Flu vaccine, please reply “1.” Reply “STOP” to opt out.”

SMS were timed to be sent before and after the scheduled WCH specialist appointments. Each child received a maximum of 3 SMS reminders. The first was sent up to 2 weeks but no less than 1 week before the appointment, the second SMS was sent 2 weeks after the first, with the third SMS sent 2 weeks later. The SMS intervention ceased if a parent replied advising that the child was immunized, and this was confirmed on the AIR. The SMS comprised (1) the influenza vaccination reminder text, (2) an option to reply if the vaccine had been received elsewhere, and (3) an option to reply STOP to opt out of receiving further SMS messages. Parents were encouraged to discuss vaccination with their child’s specialist. At the trial conclusion, both groups received an SMS message with a link to a survey.

### Data Collection

The study coordinator (K.H.) followed up all study participants. Baseline demographic information were collected for all parent-child pairs including the child’s age, sex, Aboriginal or Torres Strait Islander status, medical specialty, postcode (to determine Index of Relative Socioeconomic Disadvantage and residential location, ie, metropolitan/regional), and influenza vaccine receipt (from AIR) in 2019, 2020, and 2021. All collected identifiable data were stored on a WCHN secure database, with access to the database controlled by password protection.

### Outcomes

The primary outcome was receipt of at least 1 dose of influenza vaccine by September 30, 2021 (the end of the trial period), as confirmed on the AIR. Secondary outcomes were receipt of at least 1 dose of influenza vaccine during the optimal period (April 1-June 30) among children randomly assigned on or before June 15, and time (in days) between random assignment and receipt of vaccination. Parental acceptability of the SMS intervention was assessed.

### Sample Size

We planned to enroll at least 540 parents of children with SRMCs at the WCH. To have 80% power to detect a clinically meaningful 30% relative increase in the percentage of children vaccinated from 40% in the clinician-level (control) to 52% in the SMS intervention group, a sample size of 270 children per group was required (2-tailed α = .05).

### Statistical Analysis

All analyses were undertaken on a modified intention-to-treat basis according to the statistical analysis plan, prespecified prior to database lock (Supplement 2). For the primary outcome, the proportion of participants receiving influenza vaccination was compared between randomly assigned groups using logistic regression, with the treatment effect described using an odds ratio (OR) with a 95% CI. To aid interpretation, the difference in absolute risk of receiving the influenza vaccination between the standard and intervention groups was also derived from the logistic regression model using standardization, with a 95% CI calculated using bootstrapping. Logistic regression was also used for the secondary outcome of influenza vaccination received in the optimal period. Secondary analysis comparing time from random assignment until vaccination between the trial arms was performed using a Cox proportional hazards model and described using a hazard ratio (HR) with 95% CI. Predetermined subgroup analysis to examine effect modification of age group, pediatric subspecialty, and residential location was performed by fitting logistic regression models that included an interaction term between the treatment group and each variable. An overall test for interaction was performed, and the estimates of treatment effect (ORs) in specified subgroups and 95% CIs were presented. All regression models were adjusted for the stratification variable, age group (<5 years, 5-14 years, >14 years), which was treated as a categorical fixed effect. For participants randomly assigned in the wrong age stratum, their true age category was used rather than the randomization age category. In all analyses, a 2-sided *P* value <.05 was used to indicate statistical significance. No adjustment was made for multiple preplanned comparisons, as the overall comparison of vaccine uptake is of primary interest. Analysis was performed using R, version 4.1.1 (R Foundation for Statistical Computing).^38^

## Results

Overall, 2831 children were screened for the trial; 2212 were deemed ineligible including prior receipt of influenza vaccine before study start, leaving 619 who were randomly assigned to the intervention and control groups. After randomization, 19 participants were excluded because they had already received the 2021 influenza vaccine (n = 17) or had a medical contraindication (n = 2). A further 19 children were ineligible due to age younger than 6 months (n = 2) or 18 years or older (n = 17) at the time of randomization; however, as they were deemed otherwise eligible for influenza vaccination, they were retained in the intention-to-treat analyses.

**Figure 1.**
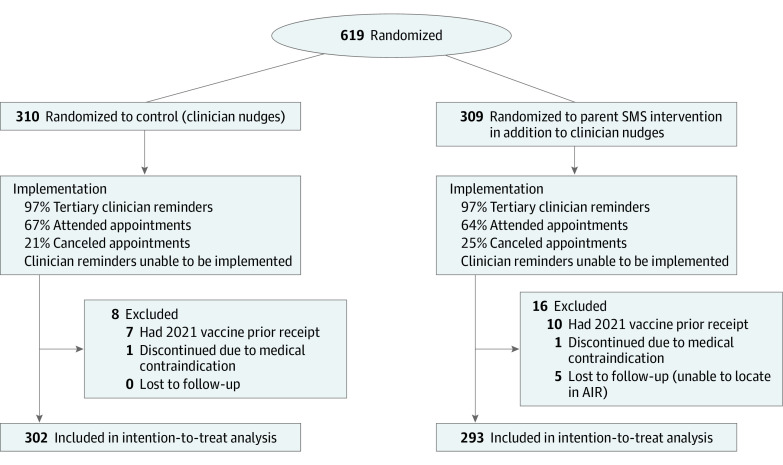
Consolidated Standards of Reporting Trials (CONSORT) Flowchart The per-protocol analysis included 293 participants from the control group and 283 participants from the short message service (SMS) intervention group (parents of children aged ≥0.5 and <18 years). AIR indicates Australian Immunisation Register.

Hence, 600 participants (intervention group: 298 [49.7%]; mean [SD] age, 11.5 [4.6] years; 162 female participants [54.4%]; 136 male participants [45.6%]; control group: 302 [50.3%]; mean [SD] age, 11.4 [4.7] years; 155 female participants [51.3%]; 147 male participants [48.7%]) completed the study. Analysis was by original assigned groups. The recruitment and randomization period was April 15 to August 13, 2021. There were no adverse events. Participants in the trial arms did not substantially differ by age, sex, Aboriginal or Torres Strait Islander or non-Indigenous status, medical risk group, Index of Relative Socioeconomic Disadvantage, or residential location. Only 12 parents (4%) opted out of the SMS messaging. Across both 2019 and 2020, there was a higher proportion of children vaccinated for influenza in the intervention group (Table 1). Overall, 199 participants (66.8%) in the control group and 189 participants (64%) in the SMS intervention group attended their appointments (Figure 1).

**Table 1.** Baseline Characteristics of Trial Participants by Randomized Arm

Characteristic	Patients, No. (%)	
	Control group	SMS intervention
No.	302	298
Child age, mean (SD), y	11.37 (4.72)	11.51 (4.59)
Child age group, y		
<5	36 (11.9)	33 (11.1)
5-14	181 (59.9)	180 (60.4)
>14	85 (28.1)	85 (28.5)
Sex		
Female	155 (51.3)	162 (54.4)
Male	147 (48.7)	136 (45.6)
Aboriginal or Torres Strait Islander status		
Aboriginal or Torres Strait Islander	17 (5.6)	14 (4.7)
Neither	248 (82.1)	250 (83.9)
Unknown	37 (12.3)	34 (11.4)
Subspecialty group		
Respiratory medicine	25 (8.3)	26 (8.7)
Neurology	86 (28.5)	81 (27.2)
Cardiology	19 (6.3)	23 (7.7)
Diabetes	113 (37.4)	93 (31.2)
Endocrine	13 (4.3)	17 (5.7)
Rheumatology clinic	33 (10.9)	45 (15.1)
Gastroenterology	13 (4.3)	13 (4.4)
Influenza vaccine receipt 2019		
Yes	115 (40.5)	129 (46.6)
Influenza vaccine receipt 2020		
Yes	118 (42.0)	134 (47.7)
IRSD quintile		
1 most disadvantaged	96 (31.8)	89 (29.9)
2	57 (18.9)	50 (16.8)
3	53 (17.5)	66 (22.1)
4	51 (16.9)	40 (13.4)
5 Least disadvantaged	45 (14.9)	53 (17.8)
Geographic region		
Regional	94 (31.1)	80 (26.8)
Metropolitan	208 (68.9)	218 (73.2)

Abbreviations: IRSD, Index of Relative Socioeconomic Disadvantage; SMS, short message service.

### Influenza Vaccine Receipt

**Figure 2.**
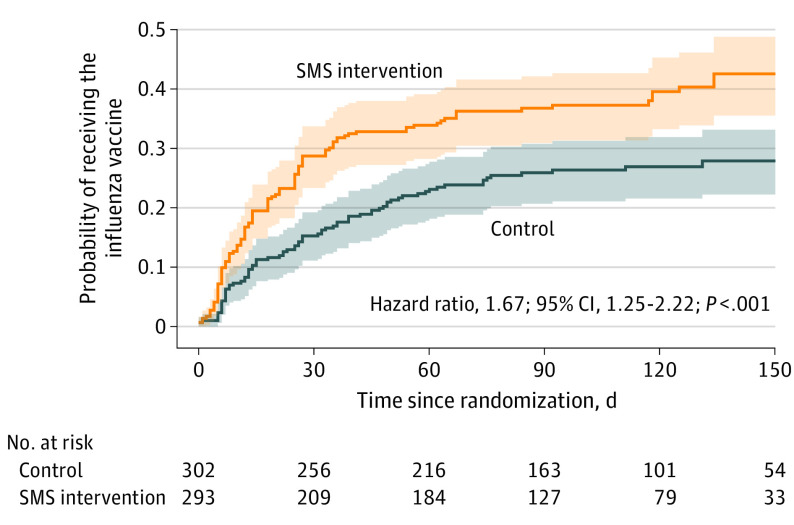
Kaplan-Meier Estimates of Time to Influenza Vaccine Receipt SMS indicates short message service.

Influenza vaccination increased from 26.2% (79 of 302) in the control group to 38.6% (113 of 293) with the SMS intervention (adjusted OR [aOR], 1.79; 95% CI, 1.27-2.55; *P* = .001) (Table 2). A similar result was observed in a sensitivity per-protocol analysis excluding 19 participants who did not meet age requirements for inclusion (aOR, 1.73; 95% CI. 1.21-2.47; *P* = .003). Time to vaccine receipt was also significantly lower in the SMS intervention group (adjusted HR [aHR], 1.67; 95% CI, 1.25-2.22; *P* < .001) (Figure 2). For the 387 participants randomly assigned before June 15, 2021, the probability of receipt in the optimal period (April 1-June 30, 2021) was higher for the SMS intervention group than in the control group (SMS group: 40.0% [76 of 190] vs 25.4% [50 of 197]; aOR, 1.97; 95% CI, 1.28-3.06; *P* = .002) (Table 2). There was little evidence that the effect of the intervention differed according to age group, subspecialty, or geographic region (eTable 1 in Supplement 3).

**Table 2.** Effect of Intervention vs Control on Probability of Receiving Influenza Vaccine in 2021

Time period	Vaccinated, No./total No. (%)		Adjusted OR (95% CI)^a^	*P* value	Risk difference (95% CI)^b^
	Control	Intervention			
2021^c^ (N = 595)	79/302 (26.2)	113/293 (38.6)	1.79 (1.27-2.55)	.001	12.6 (5.1-20.1)
During optimal period^d^ (n = 387)	50/197 (25.4)	76/190 (40.0)	1.97 (1.28-3.06)	.002	14.6 (5.6-23.8)

Abbreviation: OR, odds ratio.

^a^ Adjusted for categorical child age group (<5, 5-14, >14 years).

^b^ Risk difference derived from logistic regression model using standardization, with 95% CIs calculated using bootstrapping.

^c^ Influenza vaccine receipt until September 30, 2021.

^d^ Optimal period (defined as April 1-June 30) is restricted to participants randomly assigned on or before June 15, 2021, to allow a minimum of 2 weeks to receive vaccine. For a total of 208 participants randomly assigned after June 15, 2021, data are excluded from this analysis.

### Parent Acceptability

A parent survey was completed by 242 of 600 participants (response rate of 40.3%). Respondents’ children were more likely to have received the 2021 influenza vaccine according to the AIR compared with overall trial participants (106 of 242 [43.8%] vs 192 of 595 [32.7%]). Parents were more likely to overreport their child having received the influenza vaccination. Vaccine receipt was unable to be substantiated on the AIR for 21 of 125 children (16.8%) whose parents reported vaccine receipt, whereas 2 of 117 children (1.7%) whose parents reported not having received the vaccine were found to be vaccinated on the AIR. Parents whose children had not received the influenza vaccine were less likely to have received enough information to decide about their child receiving the vaccine compared with those whose children had received the vaccine (82 of 130 [63.1%] vs 92 of 106 [86.8%]; *P* value <.001) (eTable 2 in Supplement 3). At the time of the survey, when only children 12 years or older were eligible for the COVID-19 vaccine, 104 of 127 parents (82%) reported that their child had received it (eTable 3 in Supplement 3).

## Discussion

Results of this RCT suggest that a parent SMS nudge delivered in the context of tertiary care to children and adolescents with SRMCs increased influenza vaccine receipt. The SMS nudge intervention resulted in a more timely influenza vaccine receipt and during the optimal seasonal influenza period. This is an important finding, given the critical importance of timely influenza vaccination for this group.

This study may give policy makers and immunization program managers confidence that targeted nudge interventions can influence uptake. This is further highlighted by the fact that 20% to 25% of the children in this trial had canceled appointments or had their appointments canceled by the hospital due to COVID-19–related restrictions, thus confirming the need for strategies that increase the chances of engaging the parent and creating an opportunity to influence behavior change. During the study, all participants had access to usual care and the clinician nudges, including the opportunity to discuss the influenza vaccine with their child’s specialist, and access to the vaccine through their GP or WCH immunization clinic.

An important element of our trial design included the implementation of a waiver of consent, which used a real-world approach to ensure that all children were included in the trial. This is important, as it suggests that our findings are more likely to be representative of this cohort, which will include those who hold antivaccination views and those who are hesitant about vaccination as well as those who have positive health-seeking behaviors. It also suggests that those who do not participate in a research study may, in fact, benefit most from the nudge intervention. This was a key aspect of the trial along with the opportunity to validate influenza vaccine receipt. The wavier of consent for SMS reminders has previously been reported but not with the same effect. An RCT undertaken in the US that used a waiver of consent to implement reminders reported modest gains; however, their usual care included an automated telephone message.^35^

Our parent survey in which parents reported being more likely to be prompted through reminders from their child’s specialist as opposed to GPs confirms earlier work in which children are more likely to receive the vaccine if recommended by a specialist.^11,13^ This is likely to be a factor in the high uptake of COVID-19 vaccination in this cohort compared with overall uptake in this age group of approximately 52%.^39^ Importantly, parental acceptability of the intervention was high, and this may inform any future adaptations of the intervention. The acceptability of reminders has previously been reported, such as text messages for early childhood vaccination^40^ and postcards and interactive text messages for human papillomavirus vaccines.^41^ A study that examined 3 delivery methods of vaccination reminders for adolescent vaccination found that although postal mail reminders were preferred by most participants, text messaging and email were more effective.^42^

Our survey also showed that parents of unvaccinated children were less likely to have received enough information to decide about their child receiving the influenza vaccine. Given a lack of information has been previously identified as a barrier; this was a potentially crucial outcome. Targeted messaging, including via SMS, could be more effective and may address concerns toward the vaccine, lack of perceived influenza severity, misinformation, and negative social influences that have also been identified.^12,14,15,16,17,18,19,20,21^ However, a recent RCT undertaken in Victoria, Australia, found no difference between a motivational vs self-regulatory message for completion for human papillomavirus adolescent vaccination; yet, both were more effective than no message.^43^

### Strengths and Limitations

In addition to the wavier of consent and RCT design, the strengths of our trial included high follow-up rates and clinician-level elements that can be implemented by most health care services. A trial limitation was the inability to implement an additional nudge in the form of a reminder letter to GPs (primary care–level nudge). This was because of software that was unable to extract GP details for each patient in order for us to send reminder letters. This is an important consideration given the key role that GPs play in primary care. Additionally, the absence of a third trial arm to compare the effect of nudging patients directly (ie, without the clinician-level nudge) would have provided useful data for health systems where direct nudging of patients would be required. The research team had limited information on the parents (as nudge recipients) due to the nature of the nudge and waiver of consent; this information may have aided interpretation and identified who the nudge benefited most.

## Conclusions

Results of this RCT suggest that the addition of parent SMS nudge reminders delivered in the context of tertiary care to children and adolescents with SRMCs was superior to clinician nudges alone. This resulted in higher vaccine receipt in the optimal seasonal influenza period.

## Notes

Supplement 1. Trial Protocol

Supplement 2. Statistical Analysis Plan

Supplement 3. **eTable 1.** Effect Modification of Age Group, Subspecialty, and Geographic Region for Intervention vs Control on Probability of Receiving Influenza Vaccination in 2021 **eTable 2.** Descriptive Summary of Parent Survey Responses, Stratified by Randomized Treatment Arm, N = 242 **eTable 3.** Descriptive Summary of Parent Survey Responses to COVID-19 Vaccine-Related Questions, Stratified by Child Age Group at Time of Parent Survey, N = 234

Supplement 4. Data Sharing Statement
