# Supplementary material for: Short Message Service Reminder Nudge for Parents and Influenza Vaccination Uptake in Children and Adolescents With Special Risk Medical Conditions: The Flutext-4U Randomized Clinical Trial
Source: JAMA Pediatr. 2023 Feb 20;177(4):337–44. doi: 10.1001/jamapediatrics.2022.6145 (PMC9941970; doi:10.1001/jamapediatrics.2022.6145)
Supplement: Supplement 1. — Trial Protocol [file jamapediatr-e226145-s001.pdf]

PROTOCOL

Flutext-4U

Utilising provider-parent strategies to improve influenza vaccination in children and adolescents with special risk medical conditions: a randomised controlled trial

Protocol Version and date: Version 4.0 (13/09/2021)

Document history:

Version Number and Date	Summary of changes
2.0	Changes following HREC review.
3.0	Changes to reflect the trial running in 2021 and addition of the 'clinician reminder bookmark'.
4.0	Changes to SMS survey (additional questions) and a pre-survey primer message.

CONFIDENTIAL

This document is confidential and is the property of Women's and Children's Health Network. No part of it may be transmitted, reproduced, published, or used without prior written authorisation from the institution.

Statement of Compliance

This clinical trial will be conducted in compliance with all stipulation of this protocol, the conditions of the ethics committee approval, the National Health and Medical Research Council (NHMRC) National Statement on ethical Conduct in Human Research (2007 and all updates), the Integrated Addendum to ICH E6 (R1): Guideline for Good Clinical Practice E6 (R2), dated 9 November 2016 annotated with TGA comments and the NHMRC guidance Safety monitoring and reporting in clinical trials involving therapeutic goods (EH59, 2016).

PROTOCOL SYNOPSIS

TITLE	**Utilising provider-parent strategies to improve influenza vaccination in children and adolescents with special risk medical conditions: a randomised controlled trial**
TRIAL DESCRIPTION	A randomised trial of Flutext-4U, a multi-component intervention package which targets influenza vaccination uptake at the Parent and Provider levels.
OBJECTIVES	**Primary objective**<br>To evaluate a comprehensive multimodal strategy (Flutext-4U) to improve recommended influenza immunisation uptake in children and adolescents with medical conditions.<br>***Secondary objectives:***<br>1. Determine intervention impact by subgroup (e.g., subspecialty, age group, geographic location)<br>2. Evaluate the impact of the Flutext-4U strategy on influenza vaccination timeliness.<br>3. Determine parental acceptability of the intervention.
OUTCOMES AND OUTCOME MEASURES	**Primary outcome**<br>The proportion of medically at-risk children who receive one or more doses of influenza vaccine, assessed using the Australian Immunisation Register (AIR) records and parental report.<br><br>**Secondary outcomes:**<br>*1.* Differential intervention impact assessed by:<br>• medical risk condition<br>• age group (<5; 5-14, >14 years)<br>• geographic location (residential)<br>*2.* The proportion of medically at-risk children who receive at least one dose of influenza immunisation, as determined by AIR alone and by including parental report.<br>*3.* The proportion of children receiving immunisation during the optimal period (May to June) determined by date of vaccine receipt.<br>*4.* The difference in proportion receiving at least one dose of influenza vaccine at baseline compared to study arm#1 children (in the RCT).<br>*5.* Parental acceptability of the intervention will be assessed using SMS survey
TRIAL POPULATION	Paediatric specialists who deliver care at the Women's and Children's Hospital (WCH), medically at-risk children receiving tertiary care at the WCH who are eligible for free influenza immunisation with a hospital appointment between May and the end of July (approximately 540 children in total) and their treating GPs.
DESCRIPTION OF SITES ENROLLING PARTICIPANTS	The Women's and Children's Hospital, Adelaide.
DESCRIPTION OF INTERVENTIONS	Flutext-4U intervention<br>1. Tertiary Practice/ Provider-level interventions Provider reminders/ prompts, vaccine availability and ease of access at the WCH.<br>2. Primary Care Provider-level interventions: hard copy communication (letter) with the child's treating (referring) GP advising them that the child is identified as medically at-risk and seeking their assistance as part of the child's treating team to improve influenza vaccine uptake. Letters sent from the WCH Paediatric Outpatients Department.

	3. <u>Parent-level interventions</u> - Three SMS reminders (two-weeks apart, sent between May and the end of July. Text messages will cease once the child is immunised. Text messages will comprise: i) the influenza vaccination message reminder text, ii) an option to reply, if the vaccine has been received elsewhere and iii) an influenza vaccine decision making question.
TRIAL DURATION	The timeframe to study completion is 12 months. We anticipate a lead time for resources and testing; intervention period (May – July): identify medically at-risk children and provider prompts/ SMS messaging; and follow up to September 2021 for influenza vaccine receipt. We further anticipate 3 months for data cleaning and preliminary analysis.
PARTICIPANT DURATION	The duration of the intervention spans the 2021 influenza season. The duration for parents is from May to September 2021 , with no site study specific visits. All participating specialists at the WCH (diabetes, neurology, respiratory, gastroenterology, rheumatology, cardiology) will be exposed to the intervention. Study specialists based at the WCH and the treating GPs of children randomised to an active intervention (arm#2) will receive the practice level reminders (May until the end of July), with no site study specific visits.

Contents

PROTOCOL SYNOPSIS 2
GLOSSARY OF ABBREVIATIONS 6
INVESTIGATOR AGREEMENT 7
1. ADMINISTRATIVE INFORMATION 8
1.1. Trial registration 8
1.2. Sponsor 8
1.3. Expected duration of study 8
1.4. Stakeholder involvement 8
2. INTRODUCTION AND BACKGROUND 10
2.1. Trial rationale and aim 10
2.2. Background 10
2.3. Designing the intervention 12
2.4. Risk/Benefit assessment 14
3. TRIAL OBJECTIVES AND OUTCOMES 15
3.1. Objectives 15
3.2. Outcomes 15
4. TRIAL DESIGN 16
4.1. Overall design 16
4.2. Methods to be used to minimize bias 16
4.3. Trial population 17
4.4. Eligibility criteria 17
4.5. Screen failures 18
4.6. Identification of potential participants 18
4.7. Consent 18
5. INTERVENTION 19
5.1. Study arms 20
6. RANDOMISATION AND BLINDING 21
6.1. Concealment Mechanism 21
7. TRIAL COMPONENTS AND ASSESSMENTS 22
7.1. Schedule of Assessments 22
7.2. Description of Procedures 23
7.3. Notes on Specific Trial Visits 25
7.4. Participant Withdrawals and Losses to Follow Up 25

7.5. Trial Closure ... 26
8. DATA AND INFORMATION MANAGEMENT ... 26
8.1. Overview ... 26
8.2. Data management ... 27
9. TRIAL OVERSIGHT ... 30
9.1. Governance Structure ... 30
9.2. Study Risk Assessment and Management Plan ... 31
9.3. Quality Control and Quality Assurance ... 31
10. STATISTICAL METHODS ... 31
10.1. Sample Size Estimation ... 31
10.2. Population to Be Analysed ... 31
10.3. Methods of Analysis ... 32
10.4. Interim Analyses ... 32
11. ETHICS AND DISSEMINATION ... 32
11.1. Research Ethics Approval & Local Governance Authorisation ... 32
11.2. Amendments to the Protocol ... 32
11.3. Protocol Deviations and Serious Breaches ... 33
12. CONFIDENTIALITY ... 33
13. FINANCIAL DISCLOSURE AND CONFLICTS OF INTEREST ... 33
14. DISSEMINATION AND TRANSLATION PLAN ... 34
REFERENCES ... 35
Appendix A: Risk Assessment and Risk Management Plan ... 37

GLOSSARY OF ABBREVIATIONS

ABBREVIATION	*TERM*
AIR	Australian Immunisation Register
ATAGI	Australian Technical Advisory Group on Immunisation
CRF	Case Report Form
COSSI	Collaboration on Social Science of Immunisation
GCP	Good Clinical Practice
HREC	Human Research Ethics Committee
ITT	Intention To Treat
NHMRC	National Health and Medical Research Council
PI	Principal Investigator
QA	Quality Assurance
QC	Quality Control
RGO	Research Governance Office
SAP	Statistical Analysis Plan
SoA	Schedule of Assessments
SOP	Standard Operating Procedure
WCH	Women's and Children's Hospital

INVESTIGATOR AGREEMENT

I have read the protocol entitled "**Utilising provider-parent strategies to improve influenza vaccination in children and adolescents with special risk medical conditions: a randomised controlled trial**".

By signing this protocol, I agree to conduct the clinical trial, after approval by a Human Research Ethics Committee or Institutional Review Board (as appropriate), in accordance with the protocol, the principles of the Declaration of Helsinki and the good clinical practice guidelines adopted by the TGA [Integrated Addendum to ICH E6 (R1): Guideline for Good Clinical Practice E6 (R2), dated 9 November 2016 annotated with TGA comments].

Changes to the protocol will only be implemented after written approval is received from the Human Research Ethics Committee or Institutional Review Board (as appropriate), with the exception of medical emergencies.

I will ensure that trial staff fully understand and follow the protocol and evidence of their training is documented on the trial training log.

Name	Role	Signature and date
Prof Helen Marshall	*Sponsor-Investigator*	12.2.2020

1. ADMINISTRATIVE INFORMATION

1.1. Trial registration

1.1.1. Trial registry

The study will be registered with the Australian New Zealand Clinical Trials Registry. http://www.anzctr.org.au/

1.2. Sponsor

Trial Sponsor	Women's and Children's Health Network (WCHN)
Contact name	Prof Helen Marshall
Address	Women's and Children's Hospital<br>Kermode Street, North Adelaide<br>South Australia 5006, Australia
Sponsor-Investigator	Prof Helen Marshall

1.3. Expected duration of study

The study timeframe from identification and group allocation to study completion is 12 months. We anticipate a lead time to identify medically at-risk children, SMS messaging period (May end of July) and follow up to September 2021 for influenza vaccine receipt. We further anticipate 3 months for data cleaning and preliminary analysis.

1.4. Stakeholder involvement

To inform and optimise the design of the Flutext-4U intervention we have undertaken a rigorous development process informed by i) theoretical models; ii) a qualitative study of WCH specialist's and general practitioners interest, perceived role and delivery of influenza vaccination for children with at-risk medical conditions; iii)The Text4Health program; iv) data of emerging understanding of local barriers and solutions likely to improve vaccine receipt and v) the skills and experience of a multidisciplinary team of researchers, clinicians, social scientists and trial specialists at a preliminary workshop. Flutext-4U has been specifically designed for the Australian context, utilising parent-owned handheld devices and a communication link between the tertiary-primary care levels of care.

Additionally, other stakeholders have been consulted including:

- SA Health Immunisation Section
- Women's and Children's Health Network
- The Collaboration on Social Science of Immunisation (COSSI).

The Flutext-4U study will engage with the WCH Consumer Advisory Committee and Youth Advisory Group that includes consumer representatives, youth and other community members. This group will engage end-users support and further iterative development of the intervention components and promotion.

2. INTRODUCTION AND BACKGROUND

2.1. Trial rationale and aim

The aim of this study is to assess the effectiveness of an innovative, multi-component intervention to improve influenza vaccination in medically at-risk children and adolescents.

2.2. Background

Immunisation is an extremely successful and cost-effective public health intervention and Australia has one of the most comprehensive publicly funded immunisation programs, costing up to $500 million annually. (1) Globally, under-vaccination, particularly in specific high-risk population groups, remains a public health challenge, hampered by poor coverage data of targeted groups. (2)

Children with medical conditions are at high-risk of complications from influenza

In Australia, influenza takes more children and adolescent's lives than any other vaccine preventable disease and is the most common vaccine preventable disease requiring hospitalisation, with hospitalisation costs estimated at >$5 million per annum. (3, 4) Children and adolescents with medical conditions, as recognised in the Australian Immunisation Handbook (hereafter-referred to as high-risk children) have a significantly greater risk of influenza-associated hospitalisation and death.(5, 6) These conditions include those with chronic heart, lung, neurological, metabolic, liver or kidney diseases; cancer; diabetes; Trisomy 21 and underlying immunosuppression.(7)

The World Health Organisation (WHO) identify high-risk children as a priority group for influenza immunisation. (8) In our previous research we have shown around half of children hospitalised with influenza at the WCH have at least one high-risk condition (9), as have other authors (3) with high-risk children 30-70% more likely to be admitted to intensive care, require mechanical ventilation, develop bacterial pneumonia, have prolonged hospitalisation and die following influenza infection. (6)

Immunisation is the most effective strategy available to prevent influenza and its complications.

Individuals at highest risk of influenza-associated complications have been funded under Australia's National Immunisation Program (NIP) to receive the vaccine annually since 2010 (7, 10) with the National Seasonal Influenza Vaccination Program (NSIVP) generally commencing in the first month of autumn each year. Whilst the influenza vaccine in children can reduce the risk of influenza-associated hospitalisation including children at high-risk by 65-70% (11, 12) uptake in high-risk children is inadequate, with coverage over two consecutive years shown to be only 33%.(13) In children attending outpatient clinics reported coverage for individual years is similar across Australia at

paediatric hospitals in Adelaide (40-44%; 2014-2015)(13) Perth (50%; 2018)(14), Sydney (40%; 2011) (15), Melbourne and The Gold Coast (52%; 2017)(14).

Barriers and facilitators to influenza immunisation

Many reasons for low immunisation rates in high-risk patients are modifiable and include lack of awareness about recommendations, lack of information, not identifying children as being at risk, fear of the vaccine/side effects, inconvenience, lack of perceived severity of influenza, advised against receiving it, negative social influences, need for a priming dose in children <9-10 years perceived low efficacy of the vaccine and vaccine access problems. (15-23) Children are more likely to receive the influenza vaccine if their parents recall the child's specialist recommending it, believe that the vaccine is effective, and are not worried about adverse effects. (13, 14) Additionally, it has also been found that children are more likely to receive the vaccine if: parents have adequate awareness and knowledge, believe in the vaccine and that it works, believe it is safe, easy to access, children are younger in age (<6 years), previous vaccination, have more than one HRMC and that parents or relatives believe it is necessary along with positive social influences. (17, 20-22, 24, 25) In order to address barriers, the WCH immunisation clinic was established in 2015 to provide better access for children medically at risk and their families.

Interventions to improve influenza immunisation coverage rates

Data informing ways to overcome barriers to vaccine receipt are few. A systematic review comprising 25 studies assessing strategies to improve influenza immunisation coverage rates in children with chronic medical conditions found that interventions targeting practices, and parent or patients increased coverage by 15% (95%CI: 13-17%) and 57% (52-61%) respectively (D. Norman, unpublished data). Most studies were conducted in the US, focused only on asthmatic children and utilised traditional reminder/recall systems (e.g. letter reminders; telephone calls) that are financially costly and labour intensive. Text-message reminders sent by immunisation providers are a low-cost alternative and have been shown to increase vaccine uptake in some special-risk groups. (26-29) Notably no studies have investigated the impact of electronic reminders on influenza immunisation coverage in high-risk children.

Approach to promoting influenza immunisation in high-risk children

Despite a recommended and funded program targeting high-risk children, Australia lacks a coordinated approach to an implementation-coverage feedback loop, similar to other counties globally. National campaigns focus on pregnant women, adults with chronic health conditions and people aged >65 years who can be more readily identified. Some hospitals have established services providing immunisation, free of charge, to high-risk children. Alternatively, many hospitals and providers recommend that children attend their general practitioner for immunisation, adding to the burden of healthcare visits these families require. Our research demonstrates that

parents who receive a recommendation from their paediatrician or specialist are 4-16 times more likely to immunise their child. (13, 14) However, < 50% of parents recalled their child's paediatrician recommending influenza immunisation. (14) Current influenza immunisation promotion programs often lack consultation with providers and parents and vary significantly even between departments within the same hospital (Norman, Tuckerman unpublished data).

Multi-component interventions are optimal for improving immunisation coverage as they overcome the many direct and indirect factors that affect the vaccine decision-making process to address multiple barriers simultaneously. Based in the US, the Text4Health program implements and evaluates, using randomized control trials, tailored, targeted vaccine text message reminders, with a focus on influenza in urban paediatric and pregnant populations.(28-31) Other research targeted at the diverse and complex information needs of pregnant women and other special-risk groups (26, 27) demonstrate text-messaging interventions improve coverage. Results from a pilot cluster-randomised trial demonstrated that, women attending intervention clinics were nearly 50% more likely to receive antenatal influenza immunisation. (32) More recently, in the B Part of it study, a study lead by CI Marshall, and providing free MenB vaccine to adolescents demonstrated 97% of 34,500 adolescents returned to complete a vaccination series following SMS messaging (compared to around 90% in school HPV vaccine programs) and 88.2% completed the 12 month follow-up visit, an unprecedented achievement for a study of that size. Many barriers to influenza immunisation in high-risk children are similar to those in pregnant women and other paediatric populations. These include: i) a lack of ready access to immunisation services; ii) a lack of healthcare provider recommendation; iii) providers advising against immunisation; iv) safety concerns; v) competing priorities; vi) a lack of understanding of the need to immunise and vii) being unaware of the recommendation to immunise. (13-15, 33) A number of barriers also exist at the provider level with a sense of responsibility, knowledge and confidence of determining 'at risk' conditions, key drivers towards providing a recommendation. (CI Marshall & Tuckerman submitted manuscript) Addressing barriers at multiple levels is most likely to increase coverage in high-risk children.

2.3. Designing the intervention

Many studies, completed as part of a PhD thesis (Jane Tuckerman, The Adelaide University, 2019) conducted in medically at-risk children at the WCH, informed the design of the multi-component communication intervention package (Flutext-4U), to promote awareness, acceptance and uptake of influenza vaccination in medically at-risk children and adolescents. The thesis explored four influenza vaccination themes:

- **Impact** – The impact of influenza infection in children identified at increased risk from influenza compared to healthy children.

- **Coverage and validation** – The vaccine coverage of children identified at increased risk who are protected against seasonal influenza through vaccination and to verification of parent reported influenza vaccination compared to provider report and the Australian Immunisation Register.
- **Community awareness** – The level of parental awareness of children's influenza recommendations from a community perspective.
- **Policy and practice** – The role of children's treating general practitioners and paediatric specialists in recommending the influenza vaccine.

Synthesis of these data pertaining to South Australian children (medically at-risk) were integrated. Identified factors were mapped across the COM-B ('capability', 'opportunity', 'motivation' and 'behaviour'), a framework to understand barriers and facilitators and conceptualise interventions.(34) This subsequently helped to shape the following interventions to implement and test as part of a quality improvement initiative to improve influenza vaccination at Women's and Children's Hospital, where low rates of influenza vaccination have been identified. Several specialities will participate in this, diabetes, neurology, respiratory, gastroenterology, rheumatology and cardiology.

The **Flutext-4U** intervention package includes the following components:

1) **Tertiary-level**: Medical case note prompt/reminder stickers and clinician reminder bookmarks for WCH specialists to facilitate vaccine recommendation; vaccine availability and ease of access at the WCH.
2) **Primary Care -level**: hard copy (letter) or email communication with the child's treating (referring) GP advising them of the quality improvement initiative to improve low rates of influenza in children identified as medically at-risk and as asking them to assist as part of the child's treating team to improve influenza uptake, sent from the WCH Paediatric Outpatients Department.
3) **Parent-level:** text message reminders in a non-directive educational approach will be automatically sent to the child's parent (on behalf of the WCH) advising them that their child/adolescent is eligible for funded influenza and where they can receive it. Three SMS reminders (two-weeks apart, sent between May and the end of July. Text messages will cease once the child is immunised. Text messages will have an appropriate readability statistic score and comprise: i) the influenza vaccination message reminder text, ii) an option to reply, if the vaccine has been received elsewhere and iii) an influenza vaccine decision making question. Parents will be encouraged to engage with their child's specialist, general practitioner or immunisation provider to answer any related questions arising from the influenza vaccination message. The content of these SMS is being developed in conjunction with the WCH consumer group and Youth

Advisory Group that. Thus, although text has been established, it will be iteratively modified following engagement with these consumer groups.

2.4. Risk/Benefit assessment

2.4.1. Known potential risks

There are no risks or harms associated with drugs, procedures or devices in this study. Any risk of psychosocial distress associated with receiving a vaccine communication (SMS/letter/ reminder) or discussing vaccines is very unlikely. This study should not elevate this risk for either parents, specialists or GPs. Our research suggests both medical practitioners (sub-specialists and GPs) are open to receiving annual patient-specific reminders and numerous Australian studies indicate parents prefer influenza vaccine messaging for medically at-risk children from their child's tertiary level provider. (13-15) Any parent wanting to opt out of receiving SMS messaging can do so at any time, with no repercussion. If the intervention results in increased influenza uptake in medically at-risk children and is cost-effective, we would seek support for this to become standard care at the WCH and other paediatric hospitals in Australia.

2.4.2. Known potential benefits

It is likely that sub-specialists and GPs may benefit from receiving patient-specific reminders/prompts and that their discussions may be more detailed through application of the answers to common questions and misconceptions provided at departmental in-services and a grand-round presentation. We hope that parents may benefit from increased knowledge and awareness of the influenza recommendation and access/ availability of the vaccine.

2.4.3. Assessment of potential risks and benefits

The assessment of known potential risks and benefits of the intervention indicate negligible risk through participation in this study. Any risk of psychosocial distress associated with receiving an influenza vaccine communication (SMS/letter/ reminder) or discussing vaccines is very unlikely and is outweighed by the anticipated benefits to the individual and or knowledge that might reasonably be expected from the results.

3. TRIAL OBJECTIVES AND OUTCOMES

3.1. Objectives

3.1.1. Primary objective

To implement and evaluate a comprehensive multimodal strategy (Flutext-4U) to improve recommended influenza immunisation uptake in children and adolescents with medical conditions.

3.1.2. Secondary objectives

The secondary objectives of this trial are to determine:

1. Impact by subgroup (e.g., subspecialty, age group, residential location)
2. Parental acceptability of the SMS intervention.
3. The proportion of children receiving immunisation during the optimal period (April to June) determined by date of vaccine receipt.
4. Improvement of each intervention type, relative to baseline 2019 /2020 data.

3.1.3. Exploratory objectives

Process evaluation will assess contextual factors that may influence study outcomes, such as numbers of parents without a mobile phone number and numbers of children with an indefinite specialist referral (i.e., may not have a primary care provider).

3.2. Outcomes

3.2.1. Primary outcome

The primary outcome is receipt of one or more doses of influenza vaccine in medically at-risk children, assessed by AIR record and parental report.

3.2.2. Secondary outcomes

1. Differential intervention impact assessed by:
    - medical risk condition

- age group (<5; 5-14, >14 years)
- geographical location (residential)

2. The proportion of high-risk children who receive at least one dose of influenza immunisation, as determined by AIR alone and by including parental report (trial arm #2 only).
3. The difference in proportion receiving at least one dose of influenza vaccine at baseline compared to standard care group children (trial arm #1) or trial arm #2 using the Australian Immunisation Register to establish previous influenza vaccine receipt in 2019 and 2020.
4. The proportion of children receiving immunisation during the optimal period (April to June) determined by date of vaccine receipt.
5. Parental acceptability of the SMS intervention.

3.2.1. Process outcomes/ exploratory objectives

3.2.1.1. Contextual factors.

The numbers of parents without a mobile phone number and numbers of children with an indefinite specialist referral (i.e., may not have a primary care provider).

4. TRIAL DESIGN Overall design

The trial is a randomised controlled trial (RCT) with two parallel arms, whereby participants are randomly allocated to one of two combinations of Flutext-4U components. As text messaging incurs a small cost it is important to determine whether parent SMS messaging achieves a clinically relevant increase in uptake or if other provider or practice-based initiatives are more worthy of allocating resources.

Arm #1 (Standard care) will comprise the implementation of the tertiary practice/provider-level components: vaccine availability and ease of access at the WCH and reminders for sub-specialists (reminder prompts on medical case notes at outpatient appointments).

4.2. Methods to be used to minimize bias

Potential biases and contamination between intervention arms have been considered with strategies in place to limit any impacts. Parents allocated to each intervention arm will be recorded on a separate password protected file, only accessible to the Study Coordinator (WCHN employee). Inherently, we expect some contamination from intervention arm participants, particularly within medical risk groups due to parent interaction. Additionally, to

minimise bias at the tertiary provider level and because randomising sub-specialists would be impractical, with an almost certain chance of contamination both intervention arms will receive standard care.

4.3. Trial population

Parents of children/ adolescents with a medically at-risk condition (either diabetes, neurology, respiratory, gastroenterology, rheumatology, cardiology), their treating sub-specialist and referring GP. The study population has been chosen to be broadly representative of the target population (children and adolescents who are medically at risk for influenza) for generalisable results.

4.4. Eligibility criteria

Participants will be assigned to a randomised trial intervention only if they meet all the inclusion criteria and none of the exclusion criteria.

4.4.1. Inclusion criteria

Each participant must meet all the following criteria to be enrolled in this trial and be:

4.4.1.1. Sub-specialists & GPs

- All medical practitioners working under the diabetes, neurology, respiratory, gastroenterology, rheumatology, cardiology sub- specialities and the child's referring GP.

4.4.1.2. Parents of children / adolescents medically at risk

- Parent of a child/adolescent with a condition that is categorised as 'medically at-risk' under any of the following sub- specialities: diabetes, neurology, respiratory, gastroenterology, rheumatology, cardiology.
- Have a WCH outpatient appointment between May and the end of July 2021

4.4.2. Exclusion criteria

Patients meeting any of the following criteria will be excluded from the trial:

- Previous receipt of influenza vaccine in 2021, prior to trial commencement (defined as receipt on AIR)
- Younger sibling of another trial participant (to ensure parents are not randomised twice)

- No listed mobile phone number for parent/ guardian or have selected to not receive communication from the WCH via SMS.
- Children or adolescents with a diagnosis of Cystic Fibrosis. These children already receive additional vaccine delivery support and influenza vaccine messaging within the WCH environment.

4.5. Screen failures

Screen failures are defined as participants who are eligible to be randomised but who are found, during the screening procedures, to be ineligible to continue in the trial.

4.6. Identification of potential participants

4.6.1. Parents of children / adolescents medically at risk

We plan to enrol 540 parents. The sample size is based on the primary trial objective, influenza vaccination. The main treatment comparison of interest is the influenza vaccination receipt between trial arms. Children with specific at-risk conditions (e.g. diabetes, neurology, respiratory, gastroenterology, rheumatology, cardiology.) attending WCH specialist clinics with an appointment between May and the end of July will be identified. Lists of children with scheduled appointments will be provided from participating department specialists to the Flutext-4U Study Coordinator, a WCHN staff member.

4.7. Consent

4.7.1. Parents of children / adolescents medically at risk

A waiver of consent will be sought for parents to participate in this trial. Three criteria guide this. Firstly, the research is minimal risk. Please see risk identification and mitigation (Section 9.2). Additionally, there are plans to provide the intervention across the WCH (and potentially WCHN) if it is successful and there is insufficient evidence to determine the benefit of the intervention in the Australian context enough to threaten equipoise. Secondly, it would not be possible to obtain informed consent without threatening the validity of the trial. It would be impossible to assess SMS messaging by recruiting parents and informing them about the study and then randomising them not to receive SMS messaging. Thirdly, as annual influenza vaccination is recommended for all children/adolescents in the study, all data for the study will be collected as part of routine care (by participating specialities/departments), including patient demographics (age, gender, medical condition, postcode), specialties seen and outcomes.

5. INTERVENTION

Flutext-4U comprises elements delivered at the tertiary-level, primary care-level and parent-level.

Figure 1: Flutext-4U Intervention Components

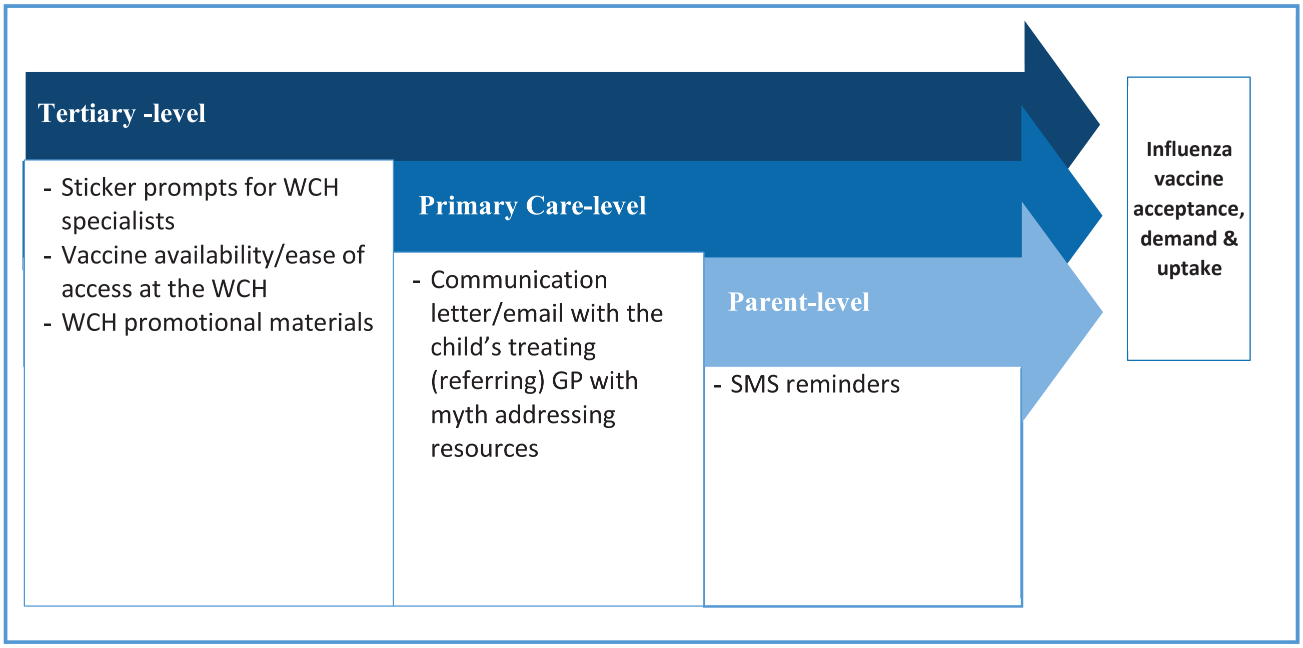

1) **Tertiary -level**: Medical case note prompt/reminder stickers and clinician reminder bookmarks for WCH specialists to facilitate vaccine recommendation; vaccine availability and ease of access at the WCH.
2) **Primary Care -level**: hard copy communication (letter/email) with the child's treating (refereeing) GP advising them of the quality improvement initiative to improve low rates of influenza in children identified as medically at-risk and as asking them to assist as part of the child's treating team to improve influenza uptake, sent from the WCH Paediatric Outpatients Department.
3) **Parent-level:** text message reminders in a non-directive educational approach will be automatically sent to the child's parent (on behalf of the WCH) advising them that their child/adolescent is eligible for funded influenza and where they can receive it. Three SMS reminders (two-weeks apart, sent between May and the end of July, timed in conjunction with the child's next WCH appointment. Text messages will cease once the child is immunised. Text messages will have an appropriate readability statistic score and comprise: i) the influenza vaccination message reminder text, ii) an option to reply, if the vaccine has been received elsewhere and iii) an influenza vaccine decision making question.

Parents will be encouraged to engage with their child's specialist, general practitioner or immunisation provider to answer any related questions arising from the influenza vaccination message.

5.1. Study arms

There are two study arms.

Arm #1 (Standard care) will comprise the implementation of some of the Tertiary Practice/Provider-level components: vaccine availability and ease of access at the WCH and reminders for sub-specialists (reminder prompts on medical case notes at outpatient appointments).

Arm #2 (All Flutext-4U intervention components) will comprise standard care (as described above) with additional Primary care practice-level reminders for GPs: hard copy communication (letter) with the child's treating (referring) GP advising them that the child is identified as qualifying for funded influenza vaccine and asking them to assist as part of the child's treating team to improve influenza uptake and Parent-level reminders (SMS messaging).

5.1.1. Trial Arm #1 - Standard Care

In 2021, as part of the overall quality improvement initiative, standard care will comprise the implementation of tertiary practice-level components: vaccine availability and ease of access at the WCH and reminders for sub-specialists. Influenza vaccine reminder stickers and clinician reminder bookmarks will be placed on the medical notes of all study participants.

5.1.2. Trial Arm #2 - All Flutext-4U intervention components

In addition to standard care, trial arm #2 will use 1) reminders for GPs and 2) parent SMS text message reminders. Reminders for GPs will comprise a hard copy communication (letter) with the child's treating (refereeing) GP advising them that the child is identified as qualifying for funded influenza vaccine and seeking them to assist as part of the child's treating team to improve vaccine uptake. Parental SMS text message reminders will be sent in a non-directive educational approach automatically to the child's parent (on behalf of the WCH) advising them that their child/adolescent is eligible for funded influenza and where they can receive it. Three SMS reminders (two-weeks apart, sent between May and the end of July. Text messages will cease once the child is immunised. Text messages will have an appropriate readability statistic score and comprise: i) the influenza vaccination message reminder text, ii) an option to reply if the vaccine has been received elsewhere. Parents will be encouraged to engage with their child's specialist, general practitioner or immunisation provider to answer any related questions arising from the influenza vaccination message.

6. RANDOMISATION AND BLINDING

Parents (of children/adolescents) will be randomised to study arm in a 1:1 ratio, using a secure web-based randomisation service; the randomisation schedule will be prepared by an independent statistician using randomly permuted blocks, stratified by age-group (<5, 5-14, >14 years). A statistician not directly involved in the analysis of the trial results will prepare the randomisation schedule. The schedule will be held by the independent statistician.

Figure 2: Flowchart of randomisation for Flutext-4U

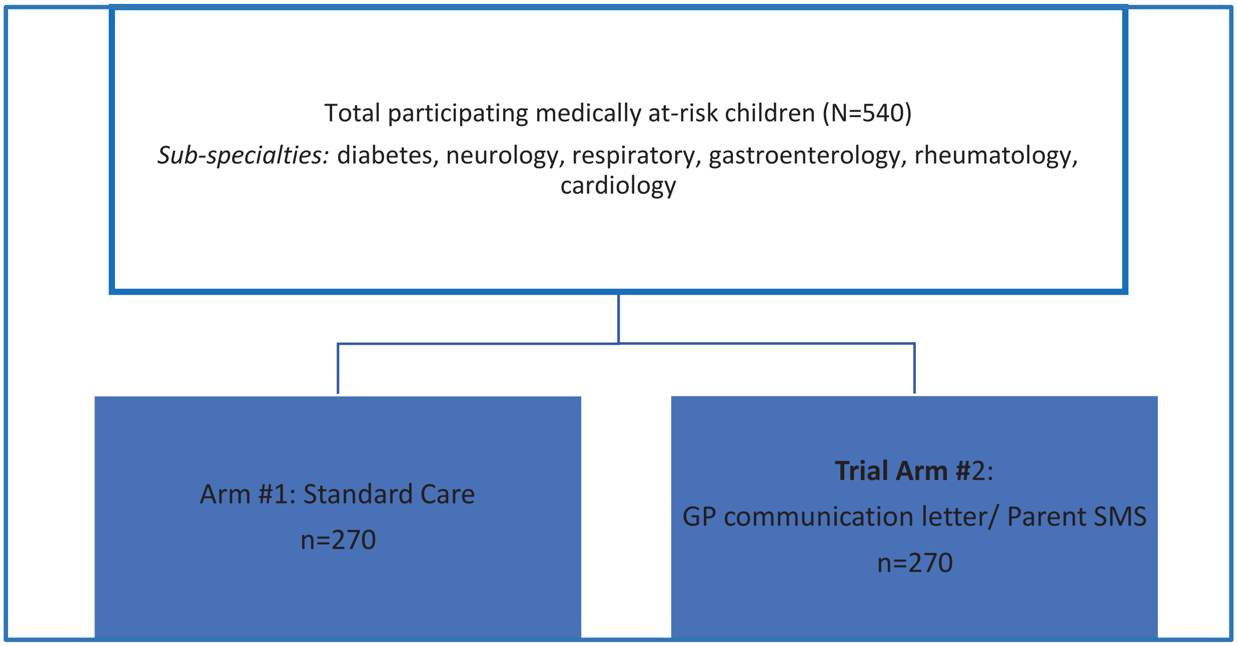

6.1. Concealment Mechanism

All children will be identified for inclusion in the study and their parents randomised once screened for inclusion. The independent statistician will create the randomisation list to assign the parents to study arms and randomisation will be concealed from the research team until after randomisation is done.

6.1.1. Blinding

Blinding is considered desirable; however, it will not be feasible to implement for the whole study population. For example, a parent who has received a SMS reminder may discuss it at the child's next specialist appointment thereby disclosing their study arm allocation.

6.1.2. WCH

Unit Heads of participating departments at the WCH will be fully informed of the nature and purpose of the trial.

6.1.3. GPs

Only GPs in trial arm #2 will receive the reminder communication letter.

6.1.4. Parents of children / adolescents medically at risk

Blinding of parents will be attempted. Some parents not receiving the parent SMS reminder (particularly within disease/condition groups) may become aware. However, they would not know the study outcomes, nor would they be aware that they are being compared.

6.1.5. Endpoint measurements and analysis

Endpoint measurements will involve low level contact of study staff, however when contact is required (such as children's AIR/ confirmation of influenza vaccine receipt etc, this will be carried out by trial staff shielded from information that might reveal trial group assignment. The study statistician undertaking analysis will remain blinded to trial intervention assignment.

7. TRIAL COMPONENTS AND ASSESSMENTS

7.1. Schedule of Assessments

**Time Point (Weeks)**	**Study Phase**	**Assessment**
Study set-up	Study set-up	• Study database set up REDCap and WCH study database.<br>• Ensure vaccine availability and ease of access at the WCH<br>• Set up of specialist prompts<br>• Set up GP contact letters<br>• Set up parent SMS
	**Flutext-4U launch**	• Departmental in-services<br>• Grand-round presentation/ launch Flutext-4U.

		• WCH signage/ promotion material – posters	
May - July	Pre-randomisation	• Identify children medically at-risk<br>• Eligibility screening<br>• Parent-child demographic data and baseline 2019/2020 influenza data obtained (AIR/ WCH records).	
	Prospective randomisation	**Intervention arm**	
		**Arm #1: Standard Care**	**Arm #2: All Flutext-4U intervention components**
	Intervention delivery	• Specialist prompts	• GP reminder letters<br>• Parent SMS (x3)
Sept	Follow-up	• Assess AIR for influenza vaccine receipt<br>• Parent survey sent via SMS to enquire if influenza vaccine received this year.	• Assess AIR for influenza vaccine receipt<br>• Parental acceptability survey via SMS text
Oct	Follow-up	• Participating specialist departments contact the children's referring GP to ascertain uptake (if not on AIR)	• Participating specialist departments contact the children's referring GP to ascertain uptake (if not on AIR)

7.2. Description of Procedures

Phase 1: Study databases will be set up. The Flutext-4U Study Coordinator will set up the system to deliver the specialist prompts (all study participants). The Study Coordinator will liaise with the WCH Outpatients Department to set up text message reminders for parents and GP reminders/ communication letters (intervention arm #2) to be sent centrally from the WCH.

Phase 2: Influenza vaccine signage to be placed around hospital.

Phase 3: Medically at-risk children will be identified and eligibility screening completed with paediatric specialists. Participants (parents) will be randomised to study arm (standard care/ intervention) and baseline demographic information will be entered for all parent-children pairs. Baseline influenza coverage for all children will be obtained from available AIR records prior to introduction of the intervention. Parent SMS reminders / specialist prompts / GP contact letters are provided, as per study trial arm. Parents may opt-out of further text messages at

any time. Parental acceptability survey is sent via SMS (both study arms). At conclusion of the trial a SMS is sent to parents in arm#1 on behalf of the WCH, to ask if the child had received an influenza vaccine in 2021.

Phase 4: Quantitative data cleaning and analysis, presentation of findings to key stakeholders to plan translation of study outcomes, final reporting and dissemination of findings.

7.2.1. Assessment procedures

7.2.1.1. Baseline demographic data

Previous studies have shown that child variables such as age and condition/s impact vaccine coverage. Additionally, socioeconomic status (SES) and geographical location and access to immunisation services have also been identified as important. Therefore, baseline demographic data collected will include the child's age, gender, medical condition, postcode (to determine SEIFA and residential location, i.e., metro/regional) and previous influenza vaccine receipt in 2019 and 2020 (from AIR records).

7.2.1.2. Uptake of influenza

Uptake of the influenza vaccine will be obtained by AIR (all participants) and parental report. Reported vaccination will also be cross-checked with AIR records. Where discrepancies are noted, WCH nursing staff from each participating department (paid using study funding) (diabetes, neurology, respiratory, gastroenterology, rheumatology, cardiology) will attempt to confirm vaccination status for any of their patients who have not received the vaccine according to AIR, with the child's referring GP (blinded to study arm).

7.2.1.1. Influenza vaccine optimal vaccine receipt

Optimal vaccine receipt will be calculated as the proportion of children receiving immunisation during the optimal period (April to June).

7.2.1.2. Parental intervention acceptability

Parents in both trial arms #1 will be invited to complete a short survey at the end of September by SMS (with a link to the questionnaire). The survey invitation message will be personalised and use the study child's first name. The reason for this is to identify the child so that questions and responses are targeted to the enrolled child. Also, following the format of the intervention SMS in which the child's first name was used.

Questions will focus on i) parental/family receipt and the COVID-19 pandemic. For parents in trial arm #2 The survey will also include questions on i) perceived benefit of SMS reminder and ii) acceptability.

A pre-survey priming message will be sent prior to the survey SMS message to inform them that the survey will follow. It is anticipated that the priming message will increase legitimacy of the message (i.e., that it is not s SPAM message) and increase survey participation.

7.3. Notes on Specific Trial Visits

7.3.1. Screening

Enrolment/Baseline of parents (Visit 1, Day 0)

- Obtain UR number, demographic information, baseline influenza vaccination history (AIR).

7.4. Participant Withdrawals and Losses to Follow Up

7.4.1. Discontinuation of treatment

Participants (Trial arm # 2 only) may discontinue the trial intervention (parental SMS reminders) for the following reasons:

- Investigator decision to discontinue a participant from the trial intervention if the participant:
    - Requires early discontinuation for reasons such as death or other serious medical complications (defined as acute severe medical illness (in the child) limiting participation in the study or child death).

The procedure for transitioning a participant off the trial intervention is as follows: notification is received from participant/ hospital department and they are removed from future trial interventions (if applicable) and assessments. A dedicated Case Report Form (CRF) page will capture the date and the specific underlying reason for discontinuation of the trial intervention. **Every attempt will be made to ensure that SMS are not sent to the parents of a deceased child. As specialist paediatricians are assisting with the study and development of the study cohort, this is very unlikely to occur.**

7.4.2. Withdrawal of consent

Participants (Trial arms # 2 only) are free to opt-out from further SMS messaging at any time upon their request or the request of their legally acceptable representative. Withdrawing from the study will not affect their access to

standard treatment or their relationship with the WCH. A dedicated Case Report Form (CRF) page will be used to capture the date of participant withdrawal.

7.5. Trial Closure

A participant is considered to have completed the trial if they have completed all phases of the trial including the last scheduled assessment (the last data collected will be the influenza vaccination status on September 30th, 2021).

The end of the trial is defined as completion of the last scheduled assessment shown in the Schedule of Assessments in the trial at all sites. The study may be temporarily suspended or prematurely terminated if there is sufficient reasonable cause (e.g. insufficient compliance to protocol requirements, data that are not sufficiently complete and/or evaluable). If the study is prematurely terminated or suspended, the Sponsor-Investigator will promptly inform HREC and RGO, the funding (where applicable) and regulatory bodies, providing the reason(s) for the termination or suspension.

8. DATA AND INFORMATION MANAGEMENT

8.1. Overview

The Principal Investigator is responsible for storing essential trial documents relevant to data management and maintaining a site-specific record of the location(s) of the site's data management-related Essential Documents. The Principal Investigator is responsible for maintaining adequate and accurate source documents that include all key observations on all participants at their site. Source data will be attributable, legible (including any changes or corrections), contemporaneous, original, accurate, complete, consistent, enduring and available. Changes to source data (hardcopy and electronic) must be traceable, must not obscure the original entry, and must be explained where this is necessary. A site-specific **Source Document Plan** will be maintained to indicate the location(s) of source documents.

The Principal Investigator will also maintain accurate case report forms (CRFs) (i.e. the data collection forms) and be responsible for ensuring that the collected and reported data is accurate, legible, complete, entered in a timely manner and enduring. To maintain the integrity of the data, any changes to data (hardcopy and electronic) must be traceable, must not obscure the original entry, and must be explained where this is necessary. Any person delegated to collect data, perform data entry or sign for data completeness will be recorded on the delegation log and will be trained to perform these trial-related duties and functions.

The Chief Investigator will be responsible for monitoring study safety and adverse events and will report any adverse events or changes to the study protocol to the HREC as required.

8.2. Data management

Generation and collection – how and data be generated and collected	**8.2.1. Data generation (source data)**<br><br>In this trial, the following types of data will be collected:<br>• personal identifying information (names, dates of birth)<br>• health data (medical condition, vaccination details)<br>• other information including vaccination views and beliefs, postcode<br><br>Source Document Plan<br>The source documents for this trial include the baseline demographic details (including baseline vaccination status) obtained by the Study Coordinator (paper); SMS responses completed by the participant (electronic); and follow up vaccination data completed by the Study Coordinator (paper). The study site will maintain a site-specific Source Document Plan that will document the source, i.e. paper, for each data discrete item/ category of items collected for the trial. This Source Document Plan, signed and dated by the Site Principal Investigator, will be prepared prior to identification of the first participant and will be filed in the site's Investigator Site File.
Generation and collection, use, storage, access and disclosure	**8.2.2. Data capture methods and data use, storage, access and disclosure during the trial**<br><br>Data collection methods<br>Data for this trial will be collected and entered using hardcopy data collection forms which will be completed by the Study Coordinator. The licensed research data collection tool REDCap will be used to collect and store all survey and non-identifiable demographic details. The exception to this is the use of the child's first name that will be used to personalise the parent SMS survey. This field will only be visible to the study team and will be deleted once data collection closes.<br><br>Use of the data<br>The data will be used for the analyses specified in the protocol and Statistical Analysis Plan.<br><br>Following the completion and analysis of the trial, the data will be retained long-term following the mandatory archive period.<br><br>Storage and access

	Hard copy data will be stored by the Site in a locked cabinet in a secure location, accessible to the research team only.<br><br>Electronic data will be securely stored in WCH database system and in files stored in WCH's network file servers, which are backed up nightly. **Files containing private or confidential data will be stored only in locations accessible only by appropriate designated members of the research team.**<br><br>REDCap is hosted on Robinson Research Institute (RRI) (The University of Adelaide) infrastructure and is subject to the same security and backup regimen as other WCH systems (e.g. the network file servers). Data is backed up nightly to a local backup server, with a monthly backup taken to tape and stored offsite. REDCap maintains an audit trail of data create/update/delete events that is accessible to project users who are granted permission to view it. Access to REDCap will be provided via a University of Adelaide user account or (for external collaborators) via a REDCap user account created by the RRI REDCAp system administrator. The permissions granted to each user within each REDCap project will be controlled by, and will be the responsibility of, the study team delegated this task by the Principal Investigator. REDCap has functionality that makes adding and removing users and managing user permissions straightforward. All data transmissions between users and the REDCap server are encrypted. The instructions for data entry to REDCap must be read and the training log signed prior to personnel commencing data entry on REDCap.<br><br>Authorised representatives of the sponsoring institution as well as representatives from the HREC, Research Governance Office and regulatory agencies may inspect all documents and records required to be maintained by the Investigator for the participants in this study. The study site will permit access to such records.<br><br>Authorised representatives of the sponsoring institution as well as representatives from the HREC, Research Governance Office and regulatory agencies may inspect all documents and records required to be maintained by the Investigator for the participants in this trial. The trial site will permit access to such records.<br><br><u>Disclosure</u><br>The trial protocol, documentation, data and all other information generated will be held in strict confidence. No information concerning the trial or the data will be released to any unauthorised third party, without prior written approval of the sponsoring institution. Clinical information will not be released without written permission of the participant, except as necessary for monitoring by the HREC, Research Governance Office or regulatory agencies.
Methods to <u>reduce</u> <u>identification</u> of participants	**8.2.3. Data confidentiality**<br><br><u>Data confidentiality</u><br>Participant confidentiality is strictly held in trust by the Site Principal Investigator, participating investigators, research staff, and the sponsoring institution and their agents.<br><br>To preserve confidentiality and reduce the risk of identification during collection, analysis and storage of data and information, the following will be undertaken:<br><br>(1) The number of private/confidential variables collected for each individual has been minimised. The data collected will be limited to that required to address the primary and secondary objectives.

	(2) Participant identifiers will be stored separately to the data collected; documents with identifiers will be stored separately to participant data. Participant data will be identified through use of a unique participant trial number/code assigned to the trial participant ("re-identifiable"). The Site Principal Investigator is responsible for the storage of a master-file of names and other identifiable data with the participant ID; access to this document will be restricted to the site trial team and authorised persons as listed previously. The master file should be stored securely, and separately, from trial data in locked/ password-protected databases with passwords kept separately.<br><br>(4) Vaccination records will be deidentified and linked to participant IDs. Survey responses will be collected online using secure REDCap software. The information linking subject IDs with personal health information or survey responses will be accessible only to the study team.<br><br>(3) Separation of the roles responsible for management of identifiers and those responsible for analysing content. The data will be analysed by the statistician, who will be provided with anonymised data identified only by the unique participant trial ID.
Quality assurance	**8.2.4. Quality assurance**<br><br>Data cleaning (e.g. checks for invalid characters, out-of-range values, invalid dates, data that is not consistent with data in other data fields, repeated participant IDs etc) will be undertaken prior to analysis, with referral to the source documents as required.<br><br>Source data verification to assess the accuracy, completeness, or representativeness of data by comparing the data in the database to the original source of the data will include a 10% random cross check, with further check as required.
Analysis	*Please refer to STATISTICS section*
Storage post-trial	**8.2.5. Archiving - Data and document retention**<br><br>Archiving<br>All data will be retained for a period of 15 years before being destroyed, in accordance with WCH policies for clinical study data.<br><br>Destruction<br>Following the archive period both data and documents will be destroyed using irreversible methods to ensure that the data is no longer usable. Hardcopies will be disposed of via a confidential shredding process.<br><br>For electronic data, software which permanently erases data will be utilised.

Data sharing	#### 8.2.6. Data sharing<br><br>Beginning 12 months following analysis and article publication, data may be made available long-term for use by future researchers from a recognised research institution whose proposed use of the data has been ethically reviewed and approved by an independent committee and who accept WCHN conditions for access.
Long-term custodianship	#### 8.2.7. Long-term custodianship (after archive period finished)<br><br>Following the conclusion of the study, all digital data will be archived in a secure WCHN, Department Paediatrics network drive which will be managed by the Chief Investigator.

9. TRIAL OVERSIGHT

9.1. Governance Structure

9.1.1. Trial Management Group (TMG)

The Site Principal Investigator is responsible for supervising any individual or party to whom they have delegated tasks at the trial site. They must provide continuous supervision and documentation of their oversight. To meet this Good Clinical Practice (GCP) requirement, a small group will be responsible for the day-to-day management of the trial and will include at a minimum the PI, study coordinator and project manager. The group will closely review all aspects of the conduct and progress of the trial, ensuring that there is a forum for identifying and addressing issues. Meetings must be minuted with attendees listed, pertinent emails retained, and phone calls documented.

9.1.2. Trial Steering Committee (TSC)

A Trial Steering Committee will be established to provide expert advice and overall supervision and ensure that the trial is conducted to the required standards. The TSC will meet at least 2-monthly, with more frequent meetings as needed, and will work to a Terms of Reference. The TSC will consist of all CIs, study coordinator and the trial statistician. This committee will review the overall conduct of the study.

9.2. Study Risk Assessment and Management Plan

A full risk assessment and management plan has been developed to manage quality throughout all stages of the trial from trial design through to reporting, with a focus on participant protection and data integrity. The plan includes identification of hazards, assessment of their risk and decisions on how to best manage and monitor risks. The plan will be reviewed on an ongoing basis during the trial. The risk assessment and management plan is documented in Appendix A of the protocol.

9.3. Quality Control and Quality Assurance

The Sponsor-Investigator will develop Standard Operating Procedures (SOPs) that identify, evaluate and control risk for all aspects of the trial, e.g. source data management, training, eligibility and informed consent. The Sponsor-Investigator will also implement quality control (QC) procedures, which will include the data entry system and data QC checks. Any missing data or data anomalies will be communicated for clarification/resolution.

10. STATISTICAL METHODS

10.1. Sample Size Estimation

We plan to enrol 540 parents of children / adolescents medically at-risk receiving tertiary care at the WCH. In order to have 80% power to detect an increase in the percentage of children vaccinated from 40% in the standard care arm to 52% in the trial arm containing all Flutext-4U components (30% relative increase; absolute increase 12%), a sample size of 270 children per group is required (two-tailed alpha = 0.05). Previous studies have shown a 30% to 70% relative increase following other immunisation interventions.

A 30% relative increase in the percentage vaccinated would be considered clinically meaningful. The world Health Organisation has called for an optimistic target of 75% coverage of influenza vaccine in medically at-risk children. We believe a realistic target for this project and confirmation of an effective strategy would be an absolute increase of at least 10% (relative increase 30%).

10.2. Population to Be Analysed

Analyses will be conducted following the intention-to-treat (ITT) principle, where outcome data are available. Participants will be compared according to the group to which they were randomly allocated, regardless of participants' compliance, crossover to other treatments or withdrawal from the trial. Thereby preserving the prognostic balance in the trial arms achieved by randomisation.

10.3. Methods of Analysis

A separate document will detail the Statistical Analysis Plan (SAP) prior to database lock. For the primary outcome, the number and proportion of participants receiving influenza vaccination in each group will be presented. The proportion vaccinated (primary outcome) will be compared between randomised groups using logistic regression, with adjustment made for variables used to stratify the randomisation. The effect of treatment will be described using an odds ratio with a 95% confidence interval. Subgroup analysis will examine the effect of medical risk condition, age group (<5, 5-14, >14 years) and geographical location (residential) on the primary outcome. Secondary analyses will be performed using logistic regression for binary outcomes and negative binomial regression for count outcomes. In all analyses, a two-sided p-value < 0.05 will be used to indicate statistical significance.

10.4. Interim Analyses

Not applicable.

11. ETHICS AND DISSEMINATION

11.1. Research Ethics Approval & Local Governance Authorisation

This protocol document and any subsequent amendments will be reviewed and approved by the human research ethics committee (HREC) prior to commencing the research. A letter of protocol approval by HREC will be obtained prior to the commencement of the trial, as well as approval for other trial documents requiring HREC review. A letter of authorisation will be obtained from the RGO prior to the commencement of the research. Institutional governance authorisation for any subsequent HREC-approved amendments will be obtained prior to implementation at each site.

11.2. Amendments to the Protocol

This trial will be conducted in compliance with the current version of the protocol. Any change to the protocol document that affects the scientific intent, trial design, participant safety, or may affect a participants willingness to continue participation in the trial is considered an amendment, and therefore will be written and filed as an amendment to this protocol and/or informed consent form. All such amendments will be submitted to the HREC, for approval prior to being implemented.

11.3. Protocol Deviations and Serious Breaches

All protocol deviations will be recorded in the participant record (source document) and on the CRF and must be reported to the Site Principal Investigator, who will assess for seriousness. Those deviations deemed to affect to a significant degree rights of a trial participant or the reliability and robustness of the data generated in the clinical trial will be reported as serious breaches. Reporting will be done in a timely manner (Site Principal Investigator to report to the Sponsor-Investigator within 72 hours and to the Site RGO within 7 day; Sponsor-Investigator to review and submit to the approving HREC within 7 days). Where protocol deviations or serious breaches identify protocol-related issues, the protocol will be reviewed and, where indicated, amended.

12. CONFIDENTIALITY

Participant confidentiality is strictly held in trust by the participating investigators, research staff, and the sponsoring institution and their agents. The trial protocol, documentation, data and all other information generated will be held in strict confidence. No information concerning the trial or the data will be released to any unauthorised third party.

Participant identifiers will be stored separately to the data collected; documents with identifiers will be stored separately to participant data. Participant data and samples will be identified through use of a unique participant trial number/code assigned to the trial participant ("re-identifiable"). The Site Principal Investigator is responsible for the storage of a master-file of names and other identifiable data with the participant ID; access to this document will be restricted to the site trial team and authorised persons as listed previously. The master file will be stored securely, and separately, from trial data in locked/ password-protected databases with passwords kept separately.

Vaccination records will be deidentified and linked to participant IDs. The information linking subject IDs with personal health information or survey responses will be accessible only to the study team. Separation of the roles responsible for management of identifiers and those responsible for analysing content. The data will be analysed by the statistician, who will be provided with anonymised data identified only by the unique participant trial ID. Clinical information will not be released without written permission of the participant, except as necessary for monitoring by HREC or regulatory agencies.

13. FINANCIAL DISCLOSURE AND CONFLICTS OF INTEREST

There are no specific declarations of interest.

14. DISSEMINATION AND TRANSLATION PLAN

Flutext-4U will be the first study to examine influenza vaccination text messaging in the context of children who are medically at-risk and has a high probability of making a significant contribution to the field. Flutext-4U addresses a major gap in our understanding of how text messaging may serve as an intermedium to mediate coverage.

This RCT, has been developed with a clear focus on high quality multidisciplinary research. Investigators, through clinical rolls and leadership of health management groups and links to the National Centre for Immunisation Research and Surveillance (NCIRS), Telethon Kids Institute, University of Western Australia, University of Melbourne, University of Adelaide and Federal and State governments are uniquely placed to ensure translation of findings into practice. This research will support changes to the way preventative care for high-risk children is managed and meet the target group's expectations of care. Increasing coverage in one of our most vulnerable populations will have a significant impact on influenza and all-cause respiratory hospitalisations. Any effects in coverage will assist other Australian jurisdictions.

Findings from the trial will be presented at national and/or international conferences related to infectious diseases, immunisation and paediatrics. The study will be written up for publication in a leading paediatric journal, such as Paediatrics, or a vaccine journal, such as Vaccine. We will share a brief summary of the findings with key stakeholders, including:

- SA Health Immunisation section
- Public Health Association Australia
- Australian Technical Advisory Group on Immunisation (ATAGI)
- The Collaboration on Social Science of Immunisation (COSSI).

REFERENCES

1. Department of Health. National Immunisation Strategy for Australia 2019–2024 [Online Report]. Canberra Australian Government; 2019 [Available from: https://beta.health.gov.au/resources/publications/national-immunisation-strategy-for-australia-2019-to-2024.
2. European Centre for Disease Prevention and Control. Seasonal influenza vaccination in Europe. Vaccination recommendations and coverage rates in the EU Member States for eight influenza seasons: 2007–2008 to 2014–2015. Stockholm: ECDC; 2017.
3. Blyth CC, Macartney,K.K., McRae,J., Clark,J., Marshall,H.S., Buttery, J., Francis, J.R., Kotsimbos,T., Kelly,P.M., Allen, A.C,. Influenza epidemiology, vaccine coverage and vaccine effectiveness in children admitted to sentinel Australian hospitals in 2017: Results from the PAEDS-FluCAN Collaboration. TO BE UPDATED. 2018.
4. Newall AT, Scuffham PA. Influenza-related disease: the cost to the Australian healthcare system. Vaccine. 2008;26(52):6818-23.
5. Gill PJ, Ashdown HF, Wang K, Heneghan C, Roberts NW, Harnden A, et al. Identification of children at risk of influenza-related complications in primary and ambulatory care: a systematic review and meta-analysis. The Lancet Respiratory medicine. 2015;3(2):139-49.
6. Tuckerman J, Misan S, Crawford NW, Marshall HS. Influenza in Children with Special Risk Medical Conditions: A Systematic Review and Meta-Analysis. PIDJ in-press. 2019.
7. Australian Technical Advisory Group on Immunisation (ATAGI). The Australian Immunisation Handbook 10th ed [Electronic book]. Canberra: Australian Government Department of Health; (2017 update) [Available from: https://immunisationhandbook.health.gov.au/.
8. World Health Organisation. Vaccines against influenza WHO position paper - November 2012. Wkly Epidemiol Rec. 2012;87(47):461-76.
9. Lakhan N, Clarke M, Mathew SM, Marshall H. Retrospective review of factors associated with severe hospitalised community-acquired influenza in a tertiary paediatric hospital in South Australia. Influenza and other respiratory viruses. 2016;10(6):479-85.
10. Australian Technical Advisory Group on Immunisation (ATAGI). Statement on the Administration of Seasonal Influenza Vaccines in 2019. Canberra: Australian Government Department of Health; 5 April 2019 [Available from: https://beta.health.gov.au/file/10519/download?token=1zBZrm6E.
11. Blyth CC, Jacoby P, Effler PV, Kelly H, Smith DW, Robins C, et al. Effectiveness of trivalent flu vaccine in healthy young children. Pediatrics. 2014;133(5):e1218-25.
12. Blyth CC, Jacoby P, Effler PV, Kelly H, Smith DW, Borland ML, et al. Influenza Vaccine Effectiveness and Uptake in Children at Risk of Severe Disease. The Pediatric infectious disease journal. 2016;35(3):309-15.
13. Tuckerman J, Misan S, Salih S, Joseph Xavier B, Crawford NW, Lynch J, et al. Influenza vaccination: Uptake and associations in a cross-sectional study of children with special risk medical conditions. Vaccine. 2018;36(52):8138-47.
14. Norman DA, Danchin M, Van Buynder P, Moore HC, Blyth CC, Seale H. Caregiver's attitudes, beliefs, and experiences for influenza vaccination in Australian children with medical comorbidities. Vaccine. 2019;37(16):2244-8.
15. Newcombe J, Kaur R, Wood N, Seale H, Palasanthiran P, Snelling T. Prevalence and determinants of influenza vaccine coverage at tertiary pediatric hospitals. Vaccine. 2014;32(48):6364-8.
16. Pandolfi E, Marino MG, Carloni E, Romano M, Gesualdo F, Borgia P, et al. The effect of physician's recommendation on seasonal influenza immunization in children with chronic diseases. BMC Public Health. 2012;12:984.(doi):10.1186/471-2458-12-984.
17. Esposito S, Marchisio P, Droghetti R, Lambertini L, Faelli N, Bosis S, et al. Influenza vaccination coverage among children with high-risk medical conditions. Vaccine. 2006;24(24):5251-5. Epub 2006 Mar 31.
18. Daley MF, Beaty BL, Barrow J, Pearson K, Crane LA, Berman S, et al. Missed opportunities for influenza vaccination in children with chronic medical conditions. Archives of pediatrics & adolescent medicine. 2005;159(10):986-91.
19. Romano M, Pandolfi E, Marino MG, Gesualdo F, Rizzo C, Carloni E, et al. Seasonal and pandemic influenza vaccine: recommendations to families of at-risk children during the 2009-10 season. Eur J Public Health. 2012;22(6):821-4. doi: 10.1093/eurpub/cks005. Epub 2012 Feb 7.

20. Gnanasekaran SK, Finkelstein JA, Hohman K, O'Brien M, Kruskal B, Lieu T. Parental perspectives on influenza vaccination among children with asthma. Public health reports. 2006;121(2):181-8.
21. Lin CJ, Zimmerman RK, Nowalk MP, Ko FS, Raymund M, Hoberman A, et al. Parental perspectives on influenza vaccination of children with chronic medical conditions. Journal of the National Medical Association. 2006;98(2):148-53.
22. Peleg N, Zevit N, Shamir R, Chodick G, Levy I. Seasonal influenza vaccination rates and reasons for non-vaccination in children with gastrointestinal disorders. Vaccine. 2015;33(1):182-6.
23. Principi N, Esposito S. Pediatric influenza prevention and control. Emerging infectious diseases. 2004;10(4):574-80.
24. Lin CJ, Nowalk MP, Zimmerman RK, Ko FS, Zoffel L, Hoberman A, et al. Beliefs and attitudes about influenza immunization among parents of children with chronic medical conditions over a two-year period. Journal of urban health : bulletin of the New York Academy of Medicine. 2006;83(5):874-83.
25. Rodriguez-Rieiro C, Dominguez-Berjon MF, Esteban-Vasallo MD, Sanchez-Perruca L, Astray-Mochales J, Fornies DI, et al. Vaccination coverage against 2009 seasonal influenza in chronically ill children and adults: analysis of population registries in primary care in Madrid (Spain). Vaccine. 2010;28(38):6203-9.
26. Stephens A, Wynn C, Stockwell M. Understanding the Use of Digital Technology to Promote Human Papillomavirus Vaccination- A RE-AIM Framework Approach. Human vaccines & immunotherapeutics. 2019.
27. Regan AK, Bloomfield L, Peters I, Effler PV. Randomized Controlled Trial of Text Message Reminders for Increasing Influenza Vaccination. Annals of family medicine. 2017;15(6):507-14.
28. Stockwell MS, Kharbanda EO, Martinez RA, Vargas CY, Vawdrey DK, Camargo S. Effect of a text messaging intervention on influenza vaccination in an urban, low-income pediatric and adolescent population: a randomized controlled trial. JAMA : the journal of the American Medical Association. 2012;307(16):1702-8.
29. Stockwell MS, Hofstetter AM, DuRivage N, Barrett A, Fernandez N, Vargas CY, et al. Text message reminders for second dose of influenza vaccine: a randomized controlled trial. Pediatrics. 2015;135(1):e83-91.
30. Stockwell MS, Kharbanda EO, Martinez RA, Lara M, Vawdrey D, Natarajan K, et al. Text4Health: impact of text message reminder-recalls for pediatric and adolescent immunizations. American journal of public health. 2012;102(2):e15-21.
31. Hofstetter AM, Vargas CY, Kennedy A, Kitayama K, Stockwell MS. Parental and provider preferences and concerns regarding text message reminder/recall for early childhood vaccinations. Preventive medicine. 2013;57(2):75-80.
32. Chamberlain AT, Seib K, Ault KA, Rosenberg ES, Frew PM, Cortes M, et al. Improving influenza and Tdap vaccination during pregnancy: A cluster-randomized trial of a multi-component antenatal vaccine promotion package in late influenza season. Vaccine. 2015;33(30):3571-9.
33. Newcombe J, Kaur R, Wood N, Seale H, Palasanthiran P, Snelling TL. Paediatrician beliefs and practices around influenza vaccination. Journal of paediatrics and child health. 2017;53(7):711-4.
34. Michie S, van Stralen MM, West R. The behaviour change wheel: a new method for characterising and designing behaviour change interventions. Implementation science : IS. 2011;6:42.

Appendix A: Risk Assessment and Risk Management Plan

**Protocol Name/No:** Utilising provider-parent strategies to improve influenza vaccination in children and adolescents with special risk medical conditions: a randomised controlled trial<br>**Protocol Version and date:** Version 3.0 (22/03/2021)	
**Investigator:** Prof Helen Marshall	**Sponsor:** Sponsor-Investigator

The following is a risk assessment and management plan throughout all stages of the trial from trial design through to reporting and reflective of the nature of the trial as behavioural intervention. It is split into 2 parts: Part 1 a risk comparison when the intervention is compared to standard care and Part 2 is an assessment of hazards and risks involved in the trial.

PART 1: RISK CATEGORY OF THE INTERVENTION [WHEN COMPARED WITH STANDARD OF CARE PRODUCT]

Flutext-4U is a behavioural intervention and as such there are no risks or harms associated with drugs, procedures or devices in this study. The assessment of known potential risks and benefits of the intervention indicate negligible risk through participation in this study. Any risk of psychosocial distress associated with receiving a vaccine communication (SMS/letter/ reminder) or discussing vaccines is very unlikely and is outweighed by the anticipated benefits to the individual and or knowledge that might reasonably be expected from the results. The risk of the intervention is comparable to standard care.

PART 2: ASSESSMENT OF HAZARDS AND RISKS INVOLVED IN THE TRIAL

HAZARDS AND RISKS OF THE INTERVENTION (Flutext-4U) AND STUDY PROCEDURES

Hazard ID	Hazard	Concerns identified<br>Provide details of trial-specific considerations / risk concerns	Hazard LIKELIHOOD of Occurrence<br>1 Remote<br>2 Unlikely<br>3 Possible<br>4 Likely<br>5 Very Likely	Hazard SEVERITY<br>1 Trivial<br>2 Minor<br>3 Moderate<br>4 Serious<br>5 Catastrophic	RISK<br>Low (1-8)<br>Medium (9-12)<br>High (15-25)	How will these risks be managed?<br>Address all concerns identified	Monitoring strategies<br>Discuss the impact on trial conduct monitoring and safety monitoring requirements
2.1 HAZARDS AND RISKS – PARTICIPANT							
1.	Potential hazards related to study intervention and/or its administration.	Intervention & administration risks:	Unlikely	Minor	Low	Written documentation of participant eligibility (to minimise ineligible participants being entered and exposed to the intervention). In the case of early trial discontinuation, a procedure and dedicated Case Report Form (CRF) will be followed to remove the participant from all pending trial encounters.	On-site monitoring of trial conduct: Eligibility (inclusion/exclusion criteria) – verification will be performed for 10% of enrolled participants.
2.	**Serious breach of protocol, ethical requirements, confidentiality**	Potential difficulties include:<br>Failure to protect participants' privacy and potentially sensitive data such as:<br>• personal identifying information (names, date of birth);<br>• health data (medical specialties seen by child, vaccination details);<br>• other information including vaccination views and beliefs<br>Collection of indirect identifiers e.g. postcode.	Unlikely	Serious	Low	A secure IT system will be used.<br>The permissions granted to each user will be controlled by, and will be the responsibility of, the trial team delegated this task by the Principal Investigator.<br>Files containing private or confidential data will be stored only in locations accessible only by appropriate designated members of the research team. Participant identifiers will be stored separately to the data collected; documents with	

Hazard ID	Hazard	Concerns identified<br>Provide details of trial-specific considerations / risk concerns	Hazard LIKELIHOOD of Occurrence<br>1 Remote<br>2 Unlikely<br>3 Possible<br>4 Likely<br>5 Very Likely	Hazard SEVERITY<br>1 Trivial<br>2 Minor<br>3 Moderate<br>4 Serious<br>5 Catastrophic	RISK<br>Low (1-8)<br>Medium (9-12)<br>High (15-25)	How will these risks be managed?<br>Address all concerns identified	Monitoring strategies<br>Discuss the impact on trial conduct monitoring and safety monitoring requirements
						identifiers will be stored separately to participant data. Participant data will be identified through use of a unique participant trial number/code assigned to the trial participant ("re-identifiable"). The Principal Investigator is responsible for the storage of a master-file of names and other identifiable data with the participant ID; access to this document will be restricted to the site trial team and authorised persons as listed previously.	
2.2 HAZARDS AND RISKS – TRIAL DESIGN, SYSTEMS, PERSONNEL & FACILITIES							
1.	**Trial inadequately powered / Poor identification of sufficient numbers**	• Insufficient power due to higher than anticipated incidence of vaccination<br>• Insufficient suitable participants to enrol<br>• Children ineligible due to vaccine receipt pre-April	Unlikely	Moderate	Low	• Reliable vaccination estimates are based on a recent WCH site survey data.<br>• Sample size calculation accounts for conservative estimates of missing data / withdrawals/ losses to follow-up.<br>• Additional medical conditions/ sub specialities may be considered if needed.	

Hazard ID	Hazard	Concerns identified<br>Provide details of trial-specific considerations / risk concerns	Hazard LIKELIHOOD of Occurrence<br>1 Remote<br>2 Unlikely<br>3 Possible<br>4 Likely<br>5 Very Likely	Hazard SEVERITY<br>1 Trivial<br>2 Minor<br>3 Moderate<br>4 Serious<br>5 Catastrophic	RISK<br>Low (1-8)<br>Medium (9-12)<br>High (15-25)	How will these risks be managed?<br>Address all concerns identified	Monitoring strategies<br>Discuss the impact on trial conduct monitoring and safety monitoring requirements
2.	**Unreliable outcome assessments of primary and main secondary outcomes**	Outcome measurements are objective.	Unlikely	Minor	Low	Vaccinations by parental report will be cross-checked with AIR records.	
3.	**Lack of robust procedure for assignment to intervention (and randomisation and blinding where applicable***)*	Participants will be randomised using computer randomisation and stratified by age.<br>The study statistician undertaking analysis will remain blinded to trial intervention assignment.<br>Given WCH specialists will be aware of the study specifics, there exists the potential for accidental unbinding to patients at outpatient appointments.	Remote	Moderate	Low	WCH specialists will be unaware which patients are allocated to which study arm, hereby removing them inadvertently revealing the group blinding, although if aware of the study they may ask if a parent has received a text reminder.	On-site monitoring of trial conduct: who undertook randomisation and allocation.
4.	**Deficiencies in intervention manufacture and/or distribution**	Various intervention component risks are as follows:<br>• GP contact letter: Incorrect address/ moved practice/ no longer practicing.<br>• SMS text messaging service: Malfunction /error with webpages (or components of).	Unlikely	Moderate	Low	Agreement will be put in place describing arrangements and responsibilities.	

Hazard ID	Hazard	Concerns identified<br>Provide details of trial-specific considerations / risk concerns	Hazard LIKELIHOOD of Occurrence<br>1 Remote<br>2 Unlikely<br>3 Possible<br>4 Likely<br>5 Very Likely	Hazard SEVERITY<br>1 Trivial<br>2 Minor<br>3 Moderate<br>4 Serious<br>5 Catastrophic	RISK<br>Low (1-8)<br>Medium (9-12)<br>High (15-25)	How will these risks be managed?<br>Address all concerns identified	Monitoring strategies<br>Discuss the impact on trial conduct monitoring and safety monitoring requirements
5.	**Poor data collection & management system**	The source documents for this trial include the baseline demographic details (including baseline vaccination status) obtained by the study coordinator (paper); SMS responses completed by the participant; and follow up vaccination data completed by the study coordinator (paper)<br>Data collection and management<br>• Quality control checks will be undertaken on a random sample of the dataset.<br>• Hard copy Case Report Forms (CRF) (i.e. data collection forms) and surveys will be used.	Unlikely	Minor	Low	• A trial procedure manual will cover data collection and management procedures.<br>• Time to entry of data from paper to database will be keep to a minimum<br>• User names and passwords will be required for secured websites and all study related documentation.	
6.	**Inexperienced and/or poorly trained personnel**	• Experience in all study phases and disease/healthcare delivery in trial area.<br>• Lack of GCP certification<br>• Lack of GCP procedures, informed consent, data confidentiality, data query management<br>• Knowledge of study procedures: trial interventions, trial investigations	Unlikely	Moderate	Low	• GCP training completed by Principal Investigator and project manager coordinator.<br>• Training will include any staff who may be involved in study procedures.<br>• Initiation procedures will also determine if adequate resources are available.	For new research staff:<br>• New staff trained in study procedures (per training logs) and delegated appropriate tasks.<br>• Ongoing review of Signature and Delegation Log, CVs, staff training logs

Hazard ID	Hazard	Concerns identified<br>Provide details of trial-specific considerations / risk concerns	Hazard LIKELIHOOD of Occurrence<br>1 Remote<br>2 Unlikely<br>3 Possible<br>4 Likely<br>5 Very Likely	Hazard SEVERITY<br>1 Trivial<br>2 Minor<br>3 Moderate<br>4 Serious<br>5 Catastrophic	RISK<br>Low (1-8)<br>Medium (9-12)<br>High (15-25)	How will these risks be managed?<br>Address all concerns identified	Monitoring strategies<br>Discuss the impact on trial conduct monitoring and safety monitoring requirements
16	**Lack of clarity regarding personnel responsibilities**	Establishment of study team roles	Unlikely	Minor	Low	All delegated tasks listed on the Signature and Delegation Log and signed by delegate and Site PI.	• Completed delegation logs<br>• Study induction logs (for evidence of trial team training)
17	**Inadequate facilities**	Potential for insufficient desk space/ computer and access to required computer software.	Unlikely	Minor	Low	Principal Investigator to ensure study coordinator has adequate area / desk space/ computer/ access to any software required.	
