# Supplementary material for: Short Message Service Reminder Nudge for Parents and Influenza Vaccination Uptake in Children and Adolescents With Special Risk Medical Conditions: The Flutext-4U Randomized Clinical Trial
Source: JAMA Pediatr. 2023 Feb 20;177(4):337–44. doi: 10.1001/jamapediatrics.2022.6145 (PMC9941970; doi:10.1001/jamapediatrics.2022.6145)
Supplement: Supplement 2. — Statistical Analysis Plan [file jamapediatr-e226145-s002.pdf]

FLUTEXT-4U TRIAL
STATISTICAL ANALYSIS PLAN (SAP)

Study Title: Utilising provider-parent strategies to improve influenza vaccination in children and adolescents with special risk medical conditions: a randomised controlled trial

Trial registration	Australian and New Zealand Clinical Trials Registry ACTRN12621000463875)
SAP version	1.0, 16 Dec 2021
SAP revision history	-
Funding	This work is supported by a Women's and Children's Hospital Foundation Grant.
SAP Author	Alana Cuthbert<br>Trial Statistician<br>SAHMRI Women & Kids, South Australian Health & Medical Research Institute

Approved by:

	Signature	***Date***
Person writing SAP	[redacted]	16 December 2021
Senior Statistician		16 December 2021
CI/Clinical Lead		16 December 2021

Table of Contents

1 PREFACE ... *3*

2 INTRODUCTION ... *3*

2.1 Background ... 3

2.2 Objectives ... 3

3 STUDY METHODS ... *3*

3.1 Study design ... 3

3.2 Randomisation ... 4

3.3 Sample size ... 4

3.4 Framework ... 4

3.5 Statistical interim analyses and stopping guidance ... 4

3.6 Timing of final analysis and unblinding ... 4

3.7 Timing of primary outcome assessment ... Error! Bookmark not defined.

4 STATISTICAL PRINCIPLES ... *5*

4.1 Confidence intervals and p values ... 5

4.2 Protocol deviations ... 5

4.3 Analysis population ... 5

5 TRIAL POPULATION ... *6*

5.1 Screening data, eligibility, recruitment, and withdrawal/follow-up ... 6

5.2 Baseline characteristics ... 6

6 ANALYSIS METHODS ... *8*

6.1 Outcome variables ... 8

6.2 Overall analysis approach ... 8

6.3 Covariate adjustment ... 8

6.4 Subgroup analyses ... 9

6.5 Methods for addressing outlying values ... 9

6.6 Methods for handling missing data ... 9

6.7 Harms ... 9

6.8 Descriptive analysis ... 10

6.9 Analysis Software ... 10

1 PREFACE

This Statistical Analysis Plan (SAP) describes the planned analyses for the Flutext-4U Trial. Any deviations from the planned analyses in this SAP will be documented with reasons in a post-analysis version of the SAP. Any post-hoc analyses not described in this SAP will also be clearly identified.

2 INTRODUCTION

2.1 Background

Children and adolescents with special risk medical conditions (SRMC) are at significantly higher risk of influenza-associated hospitalisation and death. Immunisation is the most effective strategy available to prevent influenza and its complications. Despite a comprehensive National Immunisation Program, the uptake of influenza vaccination in children and adolescents with SRMC is inadequate.

2.2 Objectives

To determine if text message reminders sent to parents of children/adolescents with SRMC increases uptake of influenza immunisation.

3 STUDY METHODS

3.1 Study design

This is a two-arm parallel group randomised controlled trial. Children and adolescents aged >6 months to <18 years with a special risk medical conditions receiving tertiary care at the Women's and Children's Hospital, South Australia, are eligible to participate (complete inclusion/exclusion criteria detailed in protocol). Parents of eligible children with SRMC are randomised to receive either standard care, or standard care plus intervention. Standard care includes prompt stickers for specialists on medical case notes and a written letter to the child's referring general practitioner. The intervention group receives standard care in addition to text message reminders from the hospital.

The primary outcome is the proportion of children/adolescents who have received at least one dose of flu vaccination between randomisation and the end of the study period (30 September 2021).

3.2 Randomisation

Parents of children/adolescents will be randomised to the standard care or intervention arm in a 1:1 ratio. The randomisation schedule will be prepared by an independent statistician and use randomly permuted blocks, stratified by age group (<5, 5-14, >14 - <18 years).

3.3 Sample size

A sample size of 540 parents of children/adolescents with SRMC (270 per group) is required to have 80% power to detect a 30% relative increase in the percentage of children vaccinated from 40% in the standard care arm to 52% in the intervention arm (two-tailed alpha = 0.05). Previous studies have shown a 30% to 70% relative increase following other immunisation interventions. A 30% relative increase in the percentage vaccinated would be considered clinically meaningful.

3.4 Framework

All comparisons will be undertaken assuming a standard superiority hypothesis testing framework.

3.5 Statistical interim analyses and stopping guidance

No interim analyses are planned.

3.6 Timing of final analysis and unblinding

The database will be locked for analysis once data collection and cleaning are complete and the SAP is approved. Following database lock, unblinded treatment groups will be made available to the trial statistician and analysis of the listed outcomes performed.

4 STATISTICAL PRINCIPLES

4.1 Confidence intervals and p values

For each outcome variable, a 95% confidence interval will be reported to express uncertainty about the estimated treatment effect. The statistical significance of the estimated treatment effect will be assessed at the 0.05 level using a two-sided comparative test. In summarising the effects of the intervention, multiple hypothesis tests will be performed due to multiple outcomes. No multiplicity adjustment will be made for the number of secondary analyses, as these are of less importance than the analysis of the primary outcome. In the absence of a formal procedure for controlling the type-I error rate, less emphasis will be placed on the results of secondary analyses.

4.2 Protocol deviations

Frequencies and percentages will be presented by randomised group for the following protocol deviations: ineligible parent/child pairs randomised; randomised in the wrong stratum; randomised to the wrong group; withdrawal from study; loss to follow-up.

4.3 Analysis population

For all outcomes, the planned analyses will be performed using an intention to treat (ITT) approach. The ITT analysis population will include all parent/child pairs, analysed as randomised, except where their exclusion criteria preclude the child receiving the influenza vaccine during follow-up. Randomised children who have a medical condition contraindicated with the flu vaccine, or have already received a flu vaccine in 2021, will be excluded from the ITT analysis. Such exclusions are not expected to bias treatment group comparisons since ineligible participants will be identified consistently and objectively using information available for all participants (e.g., previous receipt of the 2021 influenza vaccine) without knowledge of the assigned treatment group.

Descriptive statistics will also be reported using the ITT analysis population. For the primary outcome only, a sensitivity analysis will be performed in only eligible patients.

5 TRIAL POPULATION

5.1 Screening data, eligibility, recruitment, and withdrawal/follow-up

Information will be presented by treatment group (where appropriate) on the number of parent/child pairs:

- Screened
- Eligible to participate
- Ineligible to participate (by reason)
- Randomised
- Withdrawal
- With primary outcome data available

5.2 Baseline characteristics

A descriptive comparison of the randomised groups will be conducted on the baseline characteristics in Table 1. Frequencies and percentages will be reported for each variable. The clinical importance of any observed imbalances will be noted.

Residential location and Socio-Economic Indexes for Areas (SEIFA) will be obtained by linking postcode to ABS postal areas from the following datasets:

Australian Bureau of Statistics (March 2018) "Correspondence, 2017 Postcode to 2016 Remoteness Area, Table 3: Correspondence" [dataset], *1270.0.55.005 - Australian Statistical Geography Standard (ASGS): Volume 5 - Remoteness Structure, July 2016*, accessed 11 October 2021.

Australian Bureau of Statistics (March 2018) "Postal Area, Indexes, SEIFA 2016, Table 1: Postal Area (POA) SEIFA Summary, 2016" [dataset], *2033.0.55.001 - Census of Population and Housing: Socio-Economic Indexes for Areas (SEIFA), Australia, 2016*, accessed 11 October 2021.

Table 1: Baseline characteristics by treatment group

Variable	Control (n = x)	Intervention (n = y)
Age, mean (SD)		
Age group, % (n)		
<5		
5-14		
>14		
Gender, % (n)		
Female		
Male		
Ethnicity, % (n)		
Caucasian		
Aboriginal or Torres Strait Islander		
Culturally and Linguistically Diverse (CALD)		
Subspecialty, % (n)		
Respiratory Medicine		
Neurology		
Cardiology		
Diabetes		
Endocrine		
Rheumatology Clinic		
Gastroenterology		
Flu vaccine received 2019, % (n)		
Yes		
No		
Flu vaccine received 2020, % (n)		
Yes		
No		
IRSD quintile, % (n)		
1		
2		
3		
4		
5		
Residential location, % (n)		
Regional		
Metropolitan		

6 ANALYSIS METHODS

6.1 Outcome variables

1. At least one dose of flu vaccine received by child/adolescent between randomisation date and end of trial period (30 September 2021) (binary outcome – primary)
2. At least one dose of flu vaccine received by child/adolescent during the optimal period (1 April 2021 – 30 June 2021) (binary outcome – secondary)
3. Time between parent randomisation and first flu vaccination received by child/adolescent (time-to-event outcome, secondary)

6.2 Overall analysis approach

The intent is to compare the proportion of participants who received a flu vaccine between randomisation and 30 September 2021 between the standard and intervention groups. The proportion will be compared between groups using logistic regression, with the effect of treatment described as an odds ratio with 95% confidence interval. Logistic regression will also be used for the secondary outcome of flu vaccination received in the optimal period (1 April 2021 – 30 June 2021). This secondary analysis will be restricted to parent/child pairs randomised on or before 15 June 2021, to allow at least two weeks to access a flu vaccination following randomisation/intervention.

Secondary analysis comparing the time between randomisation and vaccination between standard and intervention groups will be performed using a Cox proportional hazards model, as well as compared descriptively with a plot of Kaplan-Meier survivorship estimates. The proportional hazards assumption will be checked by fitting a smoothing spline to the Schoenfeld residuals from the Cox model for each predictor and testing for a significant ($p<0.05$) relationship between residuals and failure time.

6.3 Covariate adjustment

Analyses will adjust for child age (<5, 5-14, >14 - <18) since this variable was used to stratify the randomisation. The adjustment variable will be treated as a categorical fixed effect. Where any participants have been randomised in the wrong age stratum, their true age category will be adjusted for, rather than the randomisation age category.

6.4 Subgroup analyses

Pre-specified subgroup analyses will be performed for the primary outcome to assess heterogeneity of the intervention in relation to:

1) Age group
2) Residential location (metropolitan or regional)
3) Paediatric subspecialty (diabetes/endocrine, respiratory medicine/cardiology, neurology, rheumatology clinic/gastroenterology)

As the study is not powered for subgroup analysis, this analysis is considered exploratory. Subgroup analysis will be performed by fitting a model that includes an interaction term between the treatment group and each variable. The models for residential location and paediatric subspecialty will also be adjusted for the stratification variable, age group. The estimates of treatment effect in specified subgroups and 95% confidence intervals from each model will be presented, as well as an overall test for interaction. If there is no evidence of interaction ($p>0.05$), any differences between subgroups should be regarded as due to chance.

6.5 Methods for addressing outlying values

Outliers will be queried during data collection and the statistical analysis. Unless confirmed as a data entry error, outliers will not be excluded from any analyses.

6.6 Methods for handling missing data

Missing data will be summarised descriptively by treatment group for all baseline characteristics, outcome variables and covariates for adjustment. Analyses will incorporate all available outcome data under the assumption that data are missing at random conditional on randomised group and covariates for adjustment.

6.7 Harms

No statistical analysis is planned to assess harms, as Flutext-4U is a behavioural intervention and the risk of the intervention is comparable to standard care.

6.8 Exploratory analyses

Results from parental acceptability survey will be summarised descriptively, with frequencies and percentages reported.

6.9 Analysis Software

All analyses will be performed using R version 4.1.1 (R Core Team, 2021) or later.
