# Supplementary material for: Short Message Service Reminder Nudge for Parents and Influenza Vaccination Uptake in Children and Adolescents With Special Risk Medical Conditions: The Flutext-4U Randomized Clinical Trial
Source: JAMA Pediatr. 2023 Feb 20;177(4):337–44. doi: 10.1001/jamapediatrics.2022.6145 (PMC9941970; doi:10.1001/jamapediatrics.2022.6145)
Supplement: Supplement 3. — eTable 1. Effect Modification of Age Group, Subspecialty, and Geographic Region for Intervention vs Control on Probability of Receiving Influenza Vaccination in 2021 eTable 2. Descriptive Summary of Parent Survey Responses, Stratified by Randomized Treatment Arm, N = 242 eTable 3. Descriptive Summary of Parent Survey Responses to COVID-19 Vaccine-Related Questions, Stratified by Child Age Group at Time of Parent Survey, N = 234 [file jamapediatr-e226145-s003.pdf]

Supplementary Online Content

Tuckerman J, Harper K, Sullivan TR, et al. Short message service reminder nudge for parents and influenza vaccination uptake in children and adolescents with special risk medical conditions: the Flutext-4U randomized clinical trial. *JAMA Pediatr*. Published online February 20, 2023. doi:10.1001/jamapediatrics.2022.6145

eTable 1. Effect Modification of Age Group, Subspecialty, and Geographic Region for Intervention vs Control on Probability of Receiving Influenza Vaccination in 2021

eTable 2. Descriptive Summary of Parent Survey Responses, Stratified by Randomized Treatment Arm, N = 242

eTable 3. Descriptive Summary of Parent Survey Responses to COVID-19 Vaccine-Related Questions, Stratified by Child Age Group at Time of Parent Survey, N = 234

This supplementary material has been provided by the authors to give readers additional information about their work.

© 2023 Tuckerman J et al. *JAMA Pediatrics.*

eTable 1. Effect Modification of Age Group, Subspecialty, and Geographic Region for Intervention vs Control on Probability of Receiving Influenza Vaccination in 2021

Variable	Control, n (%)	Intervention, n (%)	Adjusted Odds Ratio	95% CI	p
**Age group**					**0.95***
< 5 years	13/36 (36.1%)	16/31 (51.6%)	1.89	(0.71, 5.02)	0.20
5-14 years	46/181 (25.4%)	66/179 (36.9%)	1.71	(1.09, 2.69)	0.02
>14 years	20/85 (23.5%)	31/83 (37.3%)	1.94	(0.99, 3.79)	0.05
**Subspecialty**					**0.31***
Respiratory Medicine/Cardiology	19/44 (43.2%)	26/48 (54.2%)	1.66	(0.72, 3.81)	0.23
Neurology	23/86 (26.7%)	24/80 (30%)	1.18	(0.60, 2.33)	0.63
Diabetes/Endocrine	29/126 (23%)	39/109 (35.8%)	1.86	(1.05, 3.29)	0.03
Rheumatology/ Gastroenterology	8/46 (17.4%)	24/56 (42.9%)	3.55	(1.40, 9.01)	0.008
**Geographic region**					**0.55***
Regional	28/94 (29.8%)	37/78 (47.4%)	2.15	(1.15, 4.04)	0.02
Metropolitan	51/208 (24.5%)	76/215 (35.3%)	1.71	(1.12, 2.62)	0.01

*interaction p-value

© 2023 Tuckerman J et al. *JAMA Pediatrics.*

eTable 2. Descriptive Summary of Parent Survey Responses, Stratified by Randomized Treatment Arm, N = 242

		Control n = 130	Intervention n=112
**Question**	**Response**	**n (%)**	**n (%)**
Did your child receive the influenza vaccine this year? (n = 242)	Yes	52 (40.0)	73 (65.2)
	No	78 (60.0)	39 (34.8)
Was your child offered the influenza vaccine at a WCH hospital appointment this year? (n=239)	Yes	31 (24.0)	47 (42.7)
	No	98 (76.0)	63 (57.3)
What prompted you to get the influenza vaccine for your child this year? (n = 125)[a]	GP advice	9 (17.3)	10 (13.7)
	GP reminder	6 (11.5)	3 (4.1)
	WCH specialist advice	24 (46.2)	21 (28.8)
	WCH reminder SMS	N/A	16 (21.9)
	Another healthcare provider	3 (5.8)	2 (2.7)
	Other[#]	14 (26.9)	26 (35.6)
Did COVID-19 impact on your decision for your child to receive the influenza vaccine this year? (n=240)	Yes	33 (25.6)	26 (23.4)
	No	96 (74.4)	85 (76.6)
Where is the most convenient place for your child to receive the influenza vaccine? (n=237)	Local GP	84 (66.1)	69 (62.7)
	WCH	17 (13.4)	22 (20.0)
	Other hospital	2 (1.6)	0 (0.0)
	Pharmacy	18 (14.2)	15 (13.6)
	Other	6 (4.7)	4 (3.6)
Did you like receiving the influenza vaccine SMS reminder this year? (n=109)	Yes	N/A	67 (61.5)
	No		17 (15.6)
	No view either way		25 (22.9)
Do you want to receive an SMS reminder next year? (n=237)	Yes	75 (58.6)	78 (71.6)
	No	40 (31.2)	24 (22.0)
	Don’t know/ Can’t say	13 (10.2)	7 (6.4)
Did you have enough information to decide about your child receiving the influenza vaccine? (n=236)	Yes	88 (69.3)	86 (78.9)
	No	24 (18.9)	9 (8.3)
	Don’t know/ can’t say	15 (11.8)	14 (12.8)

Footnote: a: Responses only presented for parents who responded “Yes” to “Did your child receive the influenza vaccine this year?”

© 2023 Tuckerman J et al. *JAMA Pediatrics.*

eTable 3. Descriptive Summary of Parent Survey Responses to COVID-19 Vaccine-Related Questions, Stratified by Child Age Group at Time of Parent Survey, N = 234

Question	Response	<5 years n = 20	5-11 years n = 86	≥12 years n = 128
Has your child received a COVID-19 vaccine? (%)[a]	Yes	-	-	104 (82)
	No	-	-	23 (18)
Do you intend for your child to receive a COVID-19 vaccine? (%)[b]	Yes	7 (35)	50 (58)	7 (29)
	No	7 (35)	9 (11)	4 (17)
	Undecided	6 (30)	27 (31)	13 (54)
Would you allow your child to receive a COVID-19 vaccine if their specialist recommended it? (%)[c]	Yes	4 (31)	17 (47)	9 (53)
	No	4 (31)	3 (8)	3 (18)
	Undecided	5 (39)	16 (44)	5 (29)

Footnote: a: available data N=127; b: responses only presented for parents who did not respond “Yes” to “Has your child received a COVID-19 vaccine?” or aged <12 years [<5 years: n=20; 5-11 years: n=86; ≥12 years: n=24]; c: responses only presented for parents who did not respond “Yes” to “Do you intend for your child to receive a COVID-19 vaccine?”

© 2023 Tuckerman J et al. *JAMA Pediatrics.*
