# Supplementary material for: Short Message Service Reminder Nudge for Parents and Influenza Vaccination Uptake in Children and Adolescents With Special Risk Medical Conditions: The Flutext-4U Randomized Clinical Trial
Source: JAMA Pediatr. 2023 Feb 20;177(4):337–44. doi: 10.1001/jamapediatrics.2022.6145 (PMC9941970; doi:10.1001/jamapediatrics.2022.6145)
Supplement: Supplement 4. — Data Sharing Statement [file jamapediatr-e226145-s004.pdf]

Data Sharing Statement

Tuckerman. Short Message Service Reminder Nudge for Parents and Influenza Vaccination Uptake in Children and Adolescents With Special Risk Medical Conditions. *JAMA Pediatr*. Published February 20, 2023. doi:10.1001/jamapediatrics.2022.6145

Data
Data available: Yes
Data types: Other (please specify)
Additional Information: Trial data will be retained and archived. Trial data may be made available for use by future researchers from a recognized research institution whose proposed use of the data has been ethically reviewed and approved by an independent committee and who accept WCHN conditions for access.
How to access data: To obtain access to the data, a written proposal should be directed to the Principal Investigator, Prof Helen Marshall at the Women's and Children's Hospital, Vaccinology and Immunology Research Trials Unit via email helen.marshall@adelaide.edu.au
When available: With publication

Supporting Documents
Document types: None

Additional Information
Who can access the data: Trial data may be made available for use by future researchers from a recognized research institution whose proposed use of the data has been ethically reviewed and approved by an independent committee and who accept WCHN conditions for access.
Types of analyses: Specified purpose
Mechanisms of data availability: Signed data access agreement.
